# Tackling the Science Usability Gap in a Warming World: Co-Producing Useable Climate Information for Natural Resource Management

**DOI:** 10.1007/s00267-022-01718-4

**Published:** 2022-09-26

**Authors:** Molly S. Cross, Lauren E. Oakes, Heidi E. Kretser, Raymond Bredehoft, Paul Dey, Anika Mahoney, Noelle Smith, Ian Tator, Jim Wasseen

**Affiliations:** 1 Wildlife Conservation Society, Bronx, NY USA; 2 Department of Earth System Science, Stanford University, Stanford, CA USA; 3 Department of Natural Resources and the Environment, Cornell University, Ithaca, NY USA; 4 Wyoming Game and Fish Department, Cheyenne, WY USA

**Keywords:** Climate change, Adaptation, Natural resource management, Co-production, Actionable science

## Abstract

Developing scientific information that is used in policy and practice has been a longstanding challenge in many sectors and disciplines, including climate change adaptation for natural resource management. One approach to address this problem encourages scientists and decision-makers to co-produce usable information collaboratively. Researchers have proposed general principles for climate science co-production, yet few studies have applied and evaluated these principles in practice. In this study, climate change researchers and natural resource managers co-produced climate-related knowledge that was directly relevant for on-going habitat management planning. We documented our methods and assessed how and to what extent the process led to the near-term use of co-produced information, while also identifying salient information needs for future research. The co-production process resulted in: 1) an updated natural resource management plan that substantially differed from the former plan in how it addressed climate change, 2) increased understanding of climate change, its impacts, and management responses among agency staff, and 3) a prioritized list of climate-related information needs that would be useful for management decision-making. We found that having a boundary spanner—an intermediary with relevant science and management expertise that enables exchange between knowledge producers and users—guide the co-production process was critical to achieving outcomes. Central to the boundary spanner’s role were a range of characteristics and skills, such as knowledge of relevant science, familiarity with management issues, comfort translating science into practice, and an ability to facilitate climate-informed planning. By describing specific co-production methods and evaluating their effectiveness, we offer recommendations for others looking to co-produce climate change information to use in natural resource management planning and implementation.

## Introduction

As climate change poses increasingly unavoidable threats to human and natural systems across the planet, there is an urgent need for actions to help people, species, and ecosystems adapt to those changes (IPCC 2022). Despite a growing call for the consideration of climate change and its impacts in natural resource management and conservation practice, specifically, the use of climate-related information in decision making and on-the-ground actions remains a persistent challenge (Archie et al. 2012; Kemp et al. 2015). The difficulty of developing scientific research and knowledge that directly contributes to decision-making in policy and practice is a long-standing problem in many sectors and disciplines (Cook et al. 2013). Adding to the complexity of a general science “usability gap” (Lemos et al. 2012) are the specific challenges of using climate-related research and information. Hurdles that decision-makers commonly face when attempting to integrate climate change into planning processes include not knowing what climate information is most relevant for particular issues (Barsugli et al. 2013), challenges related to the availability, accessibility, salience, credibility, and legitimacy of available information (Moser and Ekstrom 2010), and perceptions that climate information produced by the scientific community is not useful to decisions (Archie et al. 2014; Kemp et al. 2015).

Information usability can depend on the extent and quality of interaction between information producers and users (Lemos et al. 2012). Bamzai-Dodson et al. (2021) offer a spectrum of approaches to stakeholder engagement that represents increasing levels of consultation with and participation of information users; at the highest level, stakeholders are involved in making decisions about the research. The authors suggest that there is no right or wrong level of engagement, but that the engagement approach should be aligned with project objectives, the decision context, and the nature of relationships between researchers and stakeholders. Stakeholder engagement is seen as critical to the production of actionable climate science, enabling the sourcing of research questions from practitioners and situating research and analysis within a broader planning or decision-making process (Beier et al. 2017).

Co-identifying problems and needs are key components of what some call “knowledge co-production” (Dilling and Lemos 2011), which Norström et al. (2020, p. 183) define as “iterative and collaborative processes involving diverse types of expertise, knowledge, and actors to produce context-specific knowledge”. It is a process of producing usable information through collaboration between knowledge producers and those who use knowledge to make decisions (Meadow et al. 2015). Co-production differs from a more contractual or consultative approach to science in that research questions originate from stakeholders instead of researchers, the relationship between researchers and stakeholders is a two-way partnership, and their interaction is continuous over time rather than discrete or infrequent (Mach et al. 2020). Co-production efforts can not only produce knowledge, but they can also build capacity and social capital, create and strengthen networks, and support the implementation of actions (Norström et al. 2020). Information producers and users discuss and determine expectations and goals collaboratively from the outset (Nel et al. 2016; Djenontin and Meadow 2018). Problems and questions are defined together; data may also be jointly collected and analyzed to co-create outputs (Mach et al. 2020).

In a manual for co-production specific to weather and climate services, Carter et al. (2019) indicate that intermediaries can be useful for connecting producers and users of weather and climate information. Such intermediaries may be organizations or individuals who have the time and science and policy expertise and who can utilize collaborative skill sets to apply science in a policy context (Cash et al. 2006; Briley et al. 2015; Bednarek et al. 2016). These “boundary spanners” work as science-policy intermediaries by enabling exchange between the production and use of knowledge to support evidence-informed decision-making in a specific context (Bednarek et al. 2018). Boundary spanners can take many forms, including, for example, agricultural extension agents that serve as intermediaries between universities and farmers, research programs that are embedded within resource management agencies, non-governmental organizations that facilitate the flow of information between researchers and practitioners, or collaborative partnerships that encompass information producers and users (Safford et al. 2017). Boundary spanners use a wide-array of skills, drawing upon knowledge of a particular scientific field, analytical skills to synthesize multiple lines of research, familiarity with the policy or decision-making context, communication skills for translating science, facilitation skills for coordination and building collaborations, and also working behind the scenes to connect research results and recommendations with actual decisions (Williams 2002; Bednarek et al. 2016; Jesiek et al. 2018; Goodrich et al. 2020). These skills can also reduce some of the extra time and effort costs that can be associated with knowledge co-production (Lemos et al. 2018).

Alongside growing interest in co-production approaches to incorporating climate change information into sustainability decisions, there have been calls for greater investment in evaluation of co-production processes, the use of science that is produced, and the contributions of boundary spanners (Wall et al. 2017; Posner and Cvitanovic 2019; Norström et al. 2020). Evaluations of co-production efforts are relatively rare (Lemos et al. 2018), although increasing (see for example Hyman et al. (2022)). Evaluating co-production can be challenging in part because of complexities related to evaluating both the process and outcomes; nevertheless, a range of metrics have been proposed that cover several aspects of project context and design, implementation, outputs, outcomes, and impacts (Wall et al. 2017). Outcomes and impacts criteria generally try to assess information use (Dilling and Lemos 2011), which is often categorized in terms of conceptual use (e.g., enhanced knowledge base, incorporation into planning), instrumental use (e.g., informed a new decision or action), or justification use (e.g., provided a rationale for a decision that was already made) (Pelz 1978; VanderMolen et al. 2020). Greater documentation and evaluation of real-world co-production case studies is needed to facilitate learning about what works well (or not); such information can feed back into guidance on improving the actionability of knowledge and science, including within the field of climate change-informed natural resource management (Meadow et al. 2015; Beier et al. 2017; Lemos et al. 2018).

To address this need, we conducted a case study in which scientists and natural resource managers collaboratively developed and applied a process for the co-production of climate-related knowledge that was designed to be directly applicable to habitat management planning. We documented the specific methods used to implement this co-production process and explored how the project led to near-term use of co-produced climate information, while also identifying salient, decision-relevant climate information needs for future research. Using a case study approach, we examined the following research questions in the course of trialing co-production methods and evaluating outcomes: 1) How can knowledge co-production support near-term decision-making while also identifying longer-term decision-relevant research needs? 2) In what ways can mixed methods (i.e., interviews, surveys, participatory workshops) and facilitation by a boundary spanner contribute to co-production outcomes?

We document how the co-production process evolved as it was co-developed with participating natural resource managers and assess how the process supported the use of co-produced knowledge and information. We found that the co-production process resulted in several forms of knowledge use—including the incorporation of information into an agency habitat management plan—as well as a list of climate-related research needs deemed useful to decisions being made by natural resource managers. Participating managers considered the boundary spanner to be a critical factor in achieving outcomes and articulated characteristics that contributed to the boundary spanner’s value. We offer recommendations for applying and improving co-production methods aimed at supporting climate-informed natural resource management decisions.

## Case Study Selection

We used a mixed-purposeful, opportunistic sampling approach (Patton 2002) to identify an active natural resource planning process led by an agency that was willing to embed a co-production process as a trial for integrating climate change into planning. Case study candidates were identified by a previous research project during which fish and wildlife managers at state agencies in the North Central region of the United States (Colorado, Wyoming, Montana, North Dakota, South Dakota, Kansas, and Nebraska) were asked to identify upcoming decision-making or planning opportunities for which climate change information would be useful (Crausbay and Cross 2019). During those interviews, a manager from the Wyoming Game and Fish Department (WGFD) indicated that the agency was interested in considering the effects of a changing climate in the Wyoming Statewide Habitat Plan (SHP), slated for updating in 2020. The SHP defines priorities for terrestrial and aquatic habitat protection and restoration across the state; the plan is used to coordinate conservation efforts within the agency and with external partners, and inform internal funding allocation decisions (WGFD 2020). Subsequent conversations confirmed that the agency was interested in partnering on a climate change planning experiment related to the 2020 SHP update and willing to conduct the work in a manner that would allow for learning about co-production methods and outcomes. The team leading the co-production process consisted of six staff at WGFD who were most involved in the update of the 2020 SHP (hereafter “agency core team” and also co-authors) and the lead author, who served as a boundary spanner to help design and facilitate co-production activities, shepherd the process, and connect WGFD with climate researchers from outside of the agency (hereafter “boundary spanner”). Two other co-authors advised on co-production methods, observed one project activity (a participatory workshop, described below), and conducted the evaluation of the co-production process independently from the agency core team and boundary spanner.

## Co-Production Approach

**Fig. 1:**
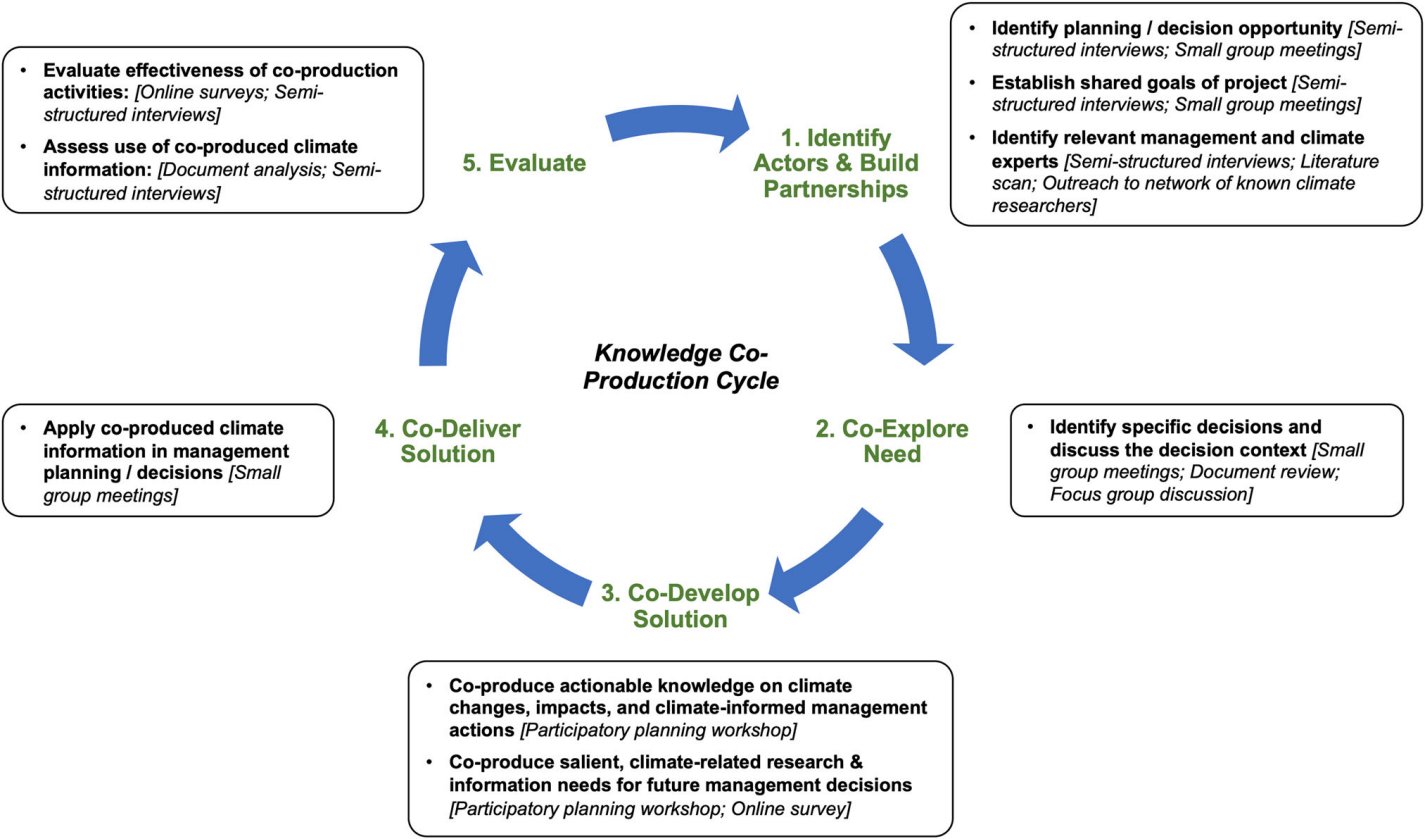
Knowledge co-production steps (green text, adapted from Vincent et al. (2018)), activities (bold text in boxes), and methods (italicized text in boxes) used in this study

We followed the co-production cycle presented by Vincent et al. (2018) which includes five steps: 1) Identify actors and build partnerships; 2) Co-explore decision needs; 3) Co-develop solution; 4) Co-deliver solution, and 5) Evaluate (Fig. 1). We also embodied the co-production principles put forth by Vincent et al. (2018): activities were co-designed using an approach that was *collaborative* and *inclusive* (i.e., information users were included in all decisions about the process), as well as *flexible* (i.e., methods were developed and modified to best fit the needs of information users); and the process was *decision-driven* (i.e., activities were designed to provide input into the update of a specific management plan), *process-based* (i.e., one stated goal was to increase the familiarity of participating managers with a process for doing climate change-informed planning) and *time-managed* (i.e., we aligned activities with the schedule for the SHP update).

We began the co-production process with tentative ideas for methods to accomplish the steps in the cycle and anticipated a mixed-methods approach that would combine small and large meetings or focus group discussions, participatory workshops, surveys, and semi-structured interviews. Method details were intentionally left open so that the agency core team and boundary spanner could collaboratively refine plans or develop new activities as needed to meet the goals of the agency and its planning process. For example, from the outset of the co-production process, we planned on holding at least one participatory workshop involving agency staff and external climate researchers, and we expected the workshop design would incorporate aspects of stepwise climate adaptation planning approaches that the boundary spanner had used in previous projects (e.g., Cross et al. 2012; Stein et al. 2014). The final plan for the workshop, however, was determined jointly by the agency core team and boundary spanner. All co-production methods and protocols were reviewed and approved by the Wildlife Conservation Society’s Institutional Review Board as they were developed.

## Co-Production Process—Methods and Results

Given the iterative, collaborative nature of this work, we present methods and results together to report how the work was conducted and what the results were for the process at various stages. As the project unfolded, the agency core team and boundary spanner co-developed and refined the co-production activities. To support this collaborative approach, the boundary spanner and agency core team met regularly via video calls (~1–2 h per month throughout the 11-month project). In this section, we focus on the methods and results for the parts of the co-production process where information was co-developed and co-applied to inform management planning (Steps 1–4, Fig. 1).

### Identify Actors and Build Partnerships

The co-production process involved three activities that served to identify actors and build partnerships: a) identifying the planning or decision-making opportunity (described in Case Study Selection), b) establishing shared goals of the project, and c) identifying relevant management and climate experts to participate in project activities. To identify shared goals for the co-production process, the boundary spanner conducted 1:1 semi-structured interviews with each member of the agency core team to ask them to articulate what benefits they were hoping to gain from being involved in the co-production effort focused on the 2020 SHP update, how they would describe success for the project, what deliverables they were hoping to gain, how they hoped the project would fit into the timeline for the SHP update, and if they knew of any climate change researchers (either within their agency or external) that were doing research that would be relevant to the SHP (see Supplementary Materials 1 for shared goals interview protocol). The boundary spanner compiled individual responses to create a list of shared goals for the project, which the agency core team and boundary spanner discussed and honed on a group call. Final goals developed for this co-production process included:

- Increase knowledge of and access to information on climate change projections, impacts, and management responses (including identifying management-relevant gaps in knowledge);
- Increase familiarity with climate-informed planning approaches;
- Improve relationships/networking with relevant climate researchers;
- Incorporate climate change into the revised Statewide Habitat Plan; and
- Advance and share learning on methods for linking climate research with natural resource management decision-making.

The agency core team identified relevant staff from WGFD to participate in co-production activities. To identify climate researchers from outside of the agency who could contribute system-specific, climate-related science and knowledge to the co-production effort, the boundary spanner used a snow-ball sampling technique (Patton 2002) that included a scan of scientific literature (i.e., peer-reviewed studies and agency science reports), interviews with the agency core team (described above), and informal conversations with climate change researchers in the region. We ultimately recruited 12 climate researchers from federal research agencies, universities, and non-governmental organizations to participate in Steps 2 & 3 (see below), most of whom described themselves as having experience conducting applied science in partnership with end users. The number of participating managers and biologists from WGFD ranged from 28 to ~40 across the various co-production steps and activities, as indicated below where appropriate. Throughout the project, we tried to refrain from characterizing participants as either “managers” vs. “scientists” (Kolstad et al. 2019), but rather considered everyone involved in the project to be “experts” who were contributing relevant knowledge, expertise, and information to the co-production process and products.

### Co-Explore Need

This step focuses on understanding the management planning and decision-making context—in our case, the SHP and management decisions that are informed by the SHP. The boundary spanner reviewed the most recent version of the plan and discussed the update process with the agency core team over the course of several meetings (all held via virtual video calls). We also held a video call between members of the agency core team and external climate researchers that were invited to participate in a climate change planning workshop (part of Step 3: Co-Develop Solution). At this meeting, WGFD staff described the priorities and information included in the SHP and the group brainstormed climate change issues likely to be of concern to focal habitats in Wyoming (see Supplementary Materials 2 for focus group discussion guide). This background on the planning and decision-making context for WGFD informed the design of future co-production activities, including the format of the participatory workshop as well as the climate change science that was gathered, summarized, and presented at the workshop.

### Co-Develop Solution

Driven by the project’s goals and WGFD’s priorities for the SHP update, this step focused on bringing together climate change researchers and WGFD managers to co-produce knowledge about: a) climate change impacts of concern to focal habitats (river, riparian, and wetland habitats); b) climate change-informed management actions of relevance to the SHP; and c) salient, climate-related information gaps to target with future research.

The core activity where management-relevant knowledge on climate change, its impacts, and climate-informed management actions was co-produced was at a participatory planning workshop (Cross et al. 2020). The workshop was held virtually, due to COVID-19 health measures, and included ~2 h of presentations covering some of the latest science on climate change and hydrological and ecological impacts and then 10 h (spread out over 2 days) of interactive workshop sessions. These interactive sessions created opportunities for all participants (WGFD staff and non-agency climate change researchers) to contribute knowledge and ideas verbally and via shared online documents on climate change impacts and vulnerabilities facing river, riparian, and wetland habitats of importance to WGFD, and climate-informed management strategies that could be included in the 2020 update of the SHP. Attendee numbers varied across the 3-day workshop but included 12 climate researchers not affiliated with WGFD who gave presentations and/or participated in the interactive workshop and at least 40 WGFD managers and biologists. Smaller breakout sessions ranged from 6–15 participants and included a mix of WGFD staff and external climate researchers. During these sessions, workshop participants co-produced a list of over 70 climate change impacts of concern to river, riparian, and wetland habitats, and more than 75 habitat management strategies and actions that could help to address climate change impacts on those focal ecosystems. The workshop agenda, breakout session worksheets, and detailed results are described in the final workshop summary report (Cross et al. 2020).

To co-produce salient, climate-related research and information needs for future management decisions by WGFD, we used a combination of discussions at the workshop and an online survey. During the workshop, participants identified 44 climate-related information gaps or needs (e.g., research, analyses, data products, inventories) that would enable the WGFD to make better climate-informed decisions about managing river, riparian, and wetland habitats in the coming years (Cross et al. 2020). To solicit further input on which of those needs were deemed by managers to be the most important gaps to fill, the agency core team and boundary spanner created an on-line survey (hereafter, “information needs survey”) that asked respondents to indicate how useful each of the 44 information needs would be to their ability to consider climate change effects in their work on river, riparian, and wetland habitats. For those information needs that were flagged as “Very Useful”, the survey included follow-up questions about how the respondent might use that information in management decisions (see Supplementary Materials 3 for information needs survey protocol). The information needs survey was sent to all WGFD staff (>350) with a 2-week turnaround window. Of those >350 people who were sent the survey, we estimate about 150–200 of those recipients would find some relevance to their work. In total, 28 WGFD staff completed the survey, representing a range of disciplines and departments within the agency. Most of the survey responses came from workshop participants (57%), although some respondents did not attend the workshop (43%). We acknowledge that this response rate is relatively low; however, we feel that the level of response is still useful for efforts by the WGFD to begin honing the full list of 44 information needs down to a shorter list that represents the most relevant information needs for management decision-making.

Each of the 44 information needs identified at the workshop had at least one survey respondent indicate that it would be “Very Useful” to their work; however, there were some information needs that were more consistently identified as being useful to WGFD staff. Eight (8) information needs were particularly useful to climate-informed habitat management efforts, with over 60% of survey respondents indicating that they were “Useful” or “Very Useful”. All but one of these highly-useful information needs were associated with at least one example of how WGFD staff would use the information in habitat management decisions, such as prioritizing habitat projects for implementation, selecting translocations sites for native species, designing culverts and other stream crossing projects, among others.

### Co-Deliver Solution

To apply the co-produced knowledge from the workshop, the boundary spanner and agency core team met regularly via video calls every 2–3 weeks to synthesize and summarize the information produced and to discuss ways that the information could be incorporated into the updated SHP. The boundary spanner led the effort to summarize workshop outputs. However, the agency core team made all decisions about how information on climate change impacts, climate-informed management actions, and climate-related knowledge gaps and needs were to be integrated into the updated SHP.

## Evaluating Co-Production Process and Outcomes—Methods and Results

To evaluate the effectiveness of the co-production activities and methods at achieving outcomes related to the near-term use of climate information (Step 5, Fig. 1), we used a mixed-methods approach involving surveys, document analysis, and semi-structured interviews. Although we used a co-production approach to design and conduct surveys related to the workshop, the document analysis and semi-structured interviews were designed and implemented by social scientists that were not involved in co-production activities (two co-authors). This research, conducted separately from co-production activities, reduced potential for bias in the document analysis and interview responses.

### Evaluation Methods

To solicit WGFD staff perspectives on the effectiveness of the participatory workshop, the boundary spanner and agency core team designed and conducted online surveys immediately before and after the workshop (hereafter “pre-workshop survey” and “post-workshop survey”). Using a mix of open- and closed-ended questions, these pre- and post-workshop surveys were designed to assess the extent to which the workshop changed participants’ knowledge about climate change and its impacts, their comfort level with integrating climate change into their work, their familiarity with climate change adaptation strategies relevant to their work, and related topics (see Supplementary Materials 4 for pre- and post-workshop survey protocols). The pre-workshop survey was sent to 65 WGFD staff that were invited to participate in the workshop; the post-workshop survey was sent to 48 WGFD staff that had indicated their intentions to participate in one or both of the participatory days of the workshop, with one reminder email. We used Welch’s two-sample t-tests to evaluate the mean change in survey responses on paired pre- and post-workshop surveys and we used linear regression to assess the relationship between individual respondents’ pre-workshop responses and their reported change after attending the workshop (see Supplementary Materials 4 for pre- and post-workshop survey analyses and results).

To evaluate the incorporation of co-produced information on climate impacts and climate-informed management actions into habitat planning by the WGFD, we conducted a document analysis of the most recent Statewide Habitat Plan, which was finalized in 2015 (2015 SHP), and the updated plan that was finalized at the end of the co-production process (2020 SHP). Using NVivo 12, we coded the two documents to assess and compare the frequency of mentions of “climate”. We carried out a content analysis surrounding any usage of the term in both documents, looking for emergent themes and determining if the integration of climate change into the planning process resulted in any shifts in mission, goals, strategies, and/or actions in 2020 SHP.

At the end of the co-production process, we conducted semi-structured interviews with each member of the agency core team (*n* = 6) and the boundary spanner (*n* = 1) to explore motivations behind the consideration of climate change in the 2020 SHP update, perceptions of the role the co-production process and the boundary spanner played in shaping any observed differences between 2015 SHP and 2020 SHP, and recommendations for others interested in doing this kind of work. We administered the interviews using an online video platform. The interview protocol consisted of 12 open-ended questions with additional prompts (see Supplementary Materials 4 for post-project interview protocol). We coded the interview data by identifying potential themes a priori from the literature on the co-production of actionable science; for example, the role of the boundary spanner and the effectiveness of the methods. We also examined emergent themes, such as key characteristics of the boundary spanner. We then analyzed each theme to better understand the perceptions of the agency core team and the boundary spanner on the co-production process and outcomes. We attribute quotes with unique identifiers in the results (M1-M6 for members of the agency core team; BS for the boundary spanner).

### Evaluation Results

#### Incorporation of Climate Change into Wyoming Statewide Habitat Plan

In our review of the 2015 SHP, we found only one mention of “climate.” In this context, climate change was mentioned as among the top five challenges facing species of conservation need and for maintaining fish and wildlife diversity across the state. Despite the recognition of climate change as a significant challenge, the plan did not directly discuss that challenge in its mission, goals, strategies, or actions.

The agency was motivated to include climate change in the 2020 SHP update for several reasons. During semi-structured interviews at the end of the project, the agency core team spoke of their constituents experiencing increased impacts from climate-related events: “*you hear more stories now from…old ranchers talking about how the winters have changed. You know, people are just having those kinds of personal experiences where they’re recognizing [climate change]”* [M4]. The agency core team also acknowledged the role of umbrella organizations such as the Association of Fish and Wildlife Agencies (AFWA) and the Western Association of Fish and Wildlife Agencies (WAFWA) recognizing climate change as an important management issue: *“…with WAFWA and all the other agencies, it’s been a hot topic”* [M1]. Some referenced the inclusion of climate change in another major planning document (the WY State Wildlife Action Plan) a few years prior as an important motivator. But the real opportunity presented itself with the boundary spanner being available and ready to work with the agency: *“…the opportunity was there with [the boundary spanner]”* [M6]. As another manager explained: *“We just felt [it was] too big, too broad, something that was a little harder to put our fingers on. But as we kind of got into it, and certainly with [the boundary spanner's] and everyone’s help, we realized that many of the things we’re already doing fall in line with actions that we can take*” [M3].

In contrast to the 2015 SHP, we found 63 mentions of climate in our review of the 2020 SHP, indicating a notable shift to address this persistent threat. Climate was the 11th most frequently used word (>3 letters), trailing behind habitat, wildlife, areas, management, priority, action, actions, biologists, fish, and project. Updates to the plan included “proactively addressing the impacts of a changing climate”. The 2020 SHP included a 3-page section devoted to climate change, comprising nearly 7% of the 44-page report; and climate change was the only habitat management threat covered by a stand-alone section. The Climate Change section detailed the importance of climate change for the state, summarized expected climate changes, and highlighted results from the aforementioned workshop. In addition to the stand-alone climate change section, the 2020 SHP also referenced climate change in the Strategies and Actions section. Throughout the plan, strategies and actions believed to be especially important to addressing climate change vulnerabilities facing fisheries and wildlife habitat were demarcated from others with a fire symbol to highlight their direct relevance to climate change adaptation. The flagging, as one agency core team manager explained, *“was a way to keep that part of our effort distinct and clear”* [M2]. Another noted: *“it was a way to message…that both new ideas and existing ideas”* [M3] for strategies and actions will be relevant in a changing climate.

We found that of the 30 strategies included in the 2020 SHP, 5 (16.7%) were flagged by the authors as important for addressing climate change and building resilience, and 1 (3.3%) explicitly mentioned climate. Of the 76 actions identified, 30 (39.5%) were similarly flagged and 8 (26.7%) explicitly mentioned climate. For example, under the strategy of promoting functional stream channels, the action of enhancing and connecting “spring creeks and cold water areas as potential cool water refugia” was flagged with a fire symbol (WGFD 2020, p. 27). As another example, under the strategy of working with landowners, land managers, and conservation organizations on grazing management, the plan emphasized new actions such as building in adaptation measures when developing grazing plans in order to account for climate change (WGFD 2020, p. 28). The agency managers that we interviewed confirmed that the final, flagged actions included in the plan emerged from the longer list generated during the workshop, which was iteratively winnowed down by the agency core team with input from other staff at WGFD to those that were deemed most relevant to and ultimately included in the 2020 SHP.

Finally, guidance for the WGFD Habitat Technical Advisory Group—which evaluates and ranks habitat project proposals to receive annual funding—was revised in 2020 to add a scoring mechanism for consideration of climate change. Under this new system, projects are rewarded in scoring if they address a specific climate change strategy or action from the 2020 SHP or otherwise identify how climate change resilience will be increased through project implementation. The agency core team managers that we interviewed noted that the final plan was not overly prescriptive in describing how point-allocation would occur in project review. However, the newly-modified system *“was a direct decision to ensure that moving forward we prioritized projects with actions that were most likely to have a positive effect in the face of climate change”* [M3].

#### Effectiveness of Co-Production Approach and Methods

The pre- and post-workshop surveys and the semi-structured interviews with the agency core team at the end of the project highlighted the value of the co-production process and methods, which allowed for group learning and processing about potential climate change impacts in the landscape and set the team up for integrating climate change into the 2020 SHP. Of the 35 agency staff that completed the post-workshop survey, 89% indicated that as a result of the workshop they: gained new knowledge about climate change projections and impacts, felt more comfortable integrating climate change information into their work, and felt more familiar with climate change adaptation strategies and actions relevant to their work. Paired survey responses from 27 individuals indicated that agency participants who came into the workshop with relatively low self-reported knowledge, comfort, or familiarity with the topic gained the most. Those who came into the workshop already knowing a fair amount, feeling fairly comfortable, and being relatively familiar with the topic reported less of a change after the workshop (see Supplementary Materials 4 for pre- and post-workshop survey analyses and results).

Interviews with the agency core team revealed additional details and opinions about the workshop’s effectiveness. One manager indicated the comprehensive approach that made the discussion tangible to participants was *“…a direct result of having…the format of the workshop…[with] experts in various climate science fields…and then having…breakout sessions where we were able to discuss”* [M4]. Here a critical piece involved making the available climate science relevant to the local context: *“Hearing climate issues at this… global level… doesn’t really seem like it has any relevance to you, but…if you can bring that data down to place and say this is what we expect to see happening, and then maybe people are… listening to that and thinking like, oh, yeah, I have seen that”* [M5]. The agency core team also emphasized the importance of including diverse perspectives: *“If we…had just…a smaller subgroup without that bigger…engagement, I don’t know if we would have gotten the same level of buy-in for incorporating climate change considerations into our management”* [M4]. Several commented on how the switch to a virtual format for the workshop enabled better participation, through use of online documents like spreadsheets that facilitated silent participation: *“I think folks that wouldn’t have spoken up otherwise wrote down ideas that were valuable ideas in those spreadsheets because it was…a safe space for everyone”* [M4]. Ultimately, the workshop provided information that was directly relevant to the 2020 SHP: *“It generated a lot of material that was really ripe for cutting and pasting and rolling into our habitat plan”* [M2].

Interviews revealed that the agency core team felt that the boundary spanner played a pivotal role in the process of integrating climate change into the 2020 SHP: *“We weren’t sure how this was going to go…[W]e hadn’t been able to do it before and being able to wrap your head around…how to do these things was a challenge. And you have a lot of different people who have a lot of different viewpoints. To get all of them to essentially agree at the end that here’s the marching orders, here’s what we’re going to do, that’s not an easy thing. And so I attribute that in large part to [the boundary spanner] and her work”* [M3]. The managers we interviewed articulated a number of characteristics for the boundary spanner that were beneficial to the process, some of which have not appeared frequently in the literature or present more detailed aspects of characteristics that have been described elsewhere (Table 1). Among those qualities, they indicated the importance of the boundary spanner making a clear time commitment, laying out specific steps for engaging staff at the workshop and soliciting their feedback in subsequent follow-up surveys, and the commitment to sorting through and assisting the core team with processing the information provided by participants. Several managers pointed out the importance of being able to ask the right questions to push the group along: *“[The boundary spanner’s] constant questioning us and asking us what we think of this and what we think of that…it was a very good process”* [M5]. Overall, the ability to drive a stepwise process captured the managers’ attention: *“…without a ‘boundary actor’ facilitator like that, we would have been hard pressed to pull that information together, to sort through it, to, you know, pick everybody’s brains and get that information into one place. I mean we just wouldn’t know where to start…if you’re not facilitating frequently it’s just kind of daunting to tackle something like that and to have somebody that works in that capacity, provide a hand…was absolutely key”* [M2].

**Table 1:** Comparison of key characteristics of a boundary spanner identified from interviews with the agency core team from Wyoming Game and Fish Department with characteristics identified in the literature; characteristics may be useful to readers seeking to employ boundary spanners to facilitate better integration of climate change information into natural resources management

Boundary spanner characteristics identified by the core agency team	Boundary spanner characteristics identified in the literature
Has charisma	Personality (e.g., Williams 2002)
Possesses relevant scientific knowledge	Knowledge of particular scientific field (e.g., Bednarek et al. 2016)
Acts as a guide	Post-modern leadership (e.g., Williams 2002)
Able to facilitate	
Asks questions	
Connects to expert speakers with relevant information for the region/local area	Integrative capacity (e.g., Bednarek et al. 2016)
Works in research and the managerial realm	
Has firm understanding of practitioner role in climate arena	
Translates research to practice	
Has access to other capacity (e.g., Ph.D. student to do a literature search)	
Provides concrete examples and experience from previous work	
*Follows-through on process and tasks*	*Not clearly represented in the reviewed literature on boundary actors and co-production*
*Keeps on an agreed timeline*	
*Frames the workshop/architecture*	
*Develops a step-by-step process*	
*Able to engage in process over time*	

All of those interviewed perceived this process as a replicable one for other agencies undergoing similar planning efforts. They noted, however, that replicability would hinge on collaboration with an effective boundary spanner: *“That whole kind of framework that we used, you could do that anywhere and just somebody to…help drive that is key”* [M2]. The boundary spanner, on the other hand, highlighted the importance of the active participation by the core agency team at WGFD: “*I found this group to be…very engaged and committed…Almost always every planning call had most if not all [members of the agency core team] participating in the discussions about the design of the workshop, giving feedback on draft materials*” [BS]. Notably, we estimate that the six members of the agency core team cumulatively spent at least 170 h on project calls and co-leading the workshop. This conservative estimate does not include additional hours reviewing workshop products and incorporating the co-produced information into the 2020 Statewide Habitat Plan; nor does it include the time that other WGFD staff spent participating in the multi-day workshop and responding to surveys. Despite that time investment, at least one member of the core agency team noted that they wished they had even more time to properly assimilate and process the information: *“I would like to [have] either more time or more people who are devoted to the process because all of us felt it was just one more thing that we had to do…I wished that I was digging in deeper because I was very interested. But just the reality of how much time I could spend on things. I didn’t feel like I always got as deep as I wanted to”* [M6].

## Discussion

Our results indicate that the co-production process and specific methods used in this study, facilitated by a knowledgeable boundary spanner, supported the near-term use of climate-related information in natural resource management planning and the identification of management-relevant climate change information needs that could guide future research. We discuss key aspects of the co-production process and boundary spanner role, detail outcomes related to the use of climate-related information and the identification of salient research needs, and offer recommendations for replicating the process.

### Key Aspects of the Co-Production Process

We employed a relatively intensive approach to end-user engagement, aligning with the “empower” category described by Bamzai-Dodson et al. (2021) where end-users are co-equal team members. The highly collaborative co-production process offered ample opportunities for the agency core team to influence the design of activities and ensure their relevance to the agency’s planning needs. This approach required that the agency invest notable staff time; over four weeks in total for the core team. The additional amount of time required to co-produce information is often highlighted (e.g., Vincent et al. 2018) and some have suggested it as a potential limit to the practicality of co-production approaches (e.g., Lemos et al. 2018). Others have shown that this time investment, measured as the frequency of meetings between researchers and users, positively affects information use (Hyman et al. 2022). In our study, the agency core team reported that the way activities were collaboratively co-designed with the boundary spanner led to a high degree of customization that allowed results to directly fit into the 2020 SHP. In addition, the involvement of a boundary spanner (see further discussion below) may have helped to offset the costs of an otherwise intensive co-production approach (Lemos et al. 2018).

Members of the agency core team valued the co-production methods used in the study for being comprehensive, structured, and locally relevant. They also appreciated that the process engaged a good number of colleagues within the agency and climate researchers from outside of the agency. They felt this participation ensured representation from a diversity of perspectives and served to increase the level of buy-in from agency staff for how climate change was ultimately included within the management plan. Although travel and meeting restrictions created by the COVID-19 pandemic required a virtual meeting rather than an in-person one, the agency core team highlighted the use of on-line, editable documents that enabled participation by relatively less vocal participants as benefits of that change. In this way, the process aligned with several tasks identified by Tengö et al. (2017) as critical for integrating multiple knowledge systems, including in this case local knowledge of natural resource managers, by facilitating the mobilization, translation, synthesis, and application of the collective knowledge of participants from varied disciplines. This inclusive approach to capturing different perspectives, ideas, and areas of expertise likely contributed to the legitimacy of the information that was co-produced, a factor that is considered critical to whether information gets used by decision-makers (Cash et al. 2003).

The co-production methods that we trialed were considered to be relevant to others working on climate change adaptation and natural resource management. The agency core team reported that the overall process and specific methods were replicable and could be useful for other agencies undergoing similar planning efforts; however, they also noted that reproducibility would likely hinge on collaboration with an effective boundary spanner.

#### Benefits of working with a boundary spanner

As has been found in other studies of co-production and actionable science (e.g., Cash et al. 2003; Bednarek et al. 2016; Goodrich et al. 2020; Jagannathan et al. 2021), the agency core team in this study considered the boundary spanner role pivotal to the co-production process’ design and success. Although the agency was motivated to consider the effects of a changing climate in their updated habitat plan, they did not have a specific plan for how to do so. Agency staff identified the boundary spanner’s ability to draw on previous experience to provide the agency with a stepwise process for getting started and to offer support throughout the planning process as key to their incorporation of climate change in the revised plan. The nature of state wildlife agency management, in which managers are spread thin with many responsibilities and limited time, suggests that it is a ripe area for harnessing collaborations with boundary spanners.

Many of the key characteristics of an effective boundary spanner identified by the agency core team echoed those from others studies, such as knowledge, charisma, an ability to act as a guide by facilitating and asking questions, cross-cultural competencies (e.g., understanding both the research and managerial realms), experience translating science into practice, and social capital that supports the involvement of and access to other experts with relevant knowledge and information (Williams 2002; Bednarek et al. 2016; Goodrich et al. 2020). However, some of the characteristics that were mentioned by WGFD staff have been less clearly articulated in the literature on boundary spanners, including practical aspects associated with the facilitating the process, such as framing an effective workshop, driving a step-by-step approach to climate-informed planning, being able to engage over an extended period of time, and timely follow-through on commitments. In these ways, the boundary spanner was able to go beyond simply playing the role of a neutral facilitator, by embodying these additional characteristics that allowed for deeper engagement and support of the planning process.

Our findings meet calls for more evidence of the benefits boundary spanners can offer (Posner and Cvitanovic 2019) and add to previous discussions of key characteristics of successful boundary spanners. Although boundary spanners can help to offset the extra time and effort that can be associated with an intensive co-production approach (Lemos et al. 2018), adequate support of boundary spanners does increase costs (Meadow et al. 2015); however, our research demonstrates the potential benefits of that investment. An improved understanding of the knowledge, skills, and traits that can support a productive boundary spanner role can help to further improve the effectiveness of these positions and organizations (Posner and Cvitanovic 2019; Goodrich et al. 2020) and hopefully lay the groundwork for increased financial support from public and private funders for this type of role to co-design and co-lead activities.

### Use of Co-Produced Climate Information

We found evidence of the use of co-produced climate change information by natural resource managers during the roughly year-long project that can be directly tied to the co-production process. This finding is notable since two challenges to evaluating the effectiveness of co-production efforts are that the use of information does not always happen immediately after it is produced and it can be difficult to attribute outcomes to particular activities (Bell et al. 2011; VanderMolen et al. 2020). The most notable use of co-produced climate change information was in the 2020 update of the Wyoming Statewide Habitat Plan (SHP), which was transformative in how it incorporated climate change. The 2020 SHP mentioned “climate” 63 times, compared to just a single mention in the 2015 version of the plan. Climate change was incorporated throughout the document and not solely discussed as a threat; numerous strategies and actions were explicitly flagged as being important to addressing climate-related threats. Perhaps most significant is the bridge that the 2020 SHP provides between *planning* and *action* on climate change, by integrating climate change into the scoring criteria that the agency uses to decide which habitat protection and restoration projects to fund each year. The new scoring system rewards projects that address climate change threats, which should help drive more agency staff to consider climate change as a standard of practice in project design and implementation. Although the decision to incorporate climate change in the 2020 SHP was motivated by pre-existing concerns within the agency, members of the agency core team indicated that they did not know how to do so and expressed that the co-production process, facilitated by a boundary spanner, was key to enabling that inclusion and that project activities and methods directly contributed to the extent to which climate change was considered.

Information use can be categorized into different types; one of those types is “conceptual use” of information, which includes informing a planning process or plan but also increasing an organization’s or individual’s understanding of a topic (Pelz 1978; VanderMolen et al. 2020). Within this case study, we documented at least two examples of conceptual information use: 1) the incorporation of co-produced climate change information in the 2020 SHP, and 2) how staff at WGFD reported feeling more knowledgeable about climate change and its impacts, more familiar with climate-informed management strategies, and more comfortable with incorporating climate change into their work as a result of participating in co-production activities (namely, the participatory climate change planning workshop). Although less visible than incorporation of information into a plan, this type of learning and change in knowledge is considered a valuable outcome of co-production efforts, with post-workshop evaluations serving as one method for measuring such learning (Meadow et al. 2015).

Conceptual information use can be a precursor to later “instrumental use” where information is directly incorporated into decisions or actions (Pelz 1978; VanderMolen et al. 2020). In an analysis of research projects funded by the US Geological Survey’s Southeast Climate Adaptation Science Center, Hyman et al. (2022) found that conceptual information uses directly influenced the level of instrumental uses. Although we did not document any direct examples of instrumental use resulting from this project, the SHP is actively used by WGFD to make decisions, including about which on-the-ground habitat projects should receive funding during an annual allocation process. Therefore, the incorporation of co-produced climate change information in the 2020 SHP may serve as a step towards instrumental use. In particular, the incorporation of a new scoring mechanism that rewards projects that address climate change could create an incentive for moving the plan into action. Future research could track additional ways the agency or partners use the climate change information co-produced in this project.

### Identification of Salient, Climate-Related Information Needs

The co-production process described in this case study successfully identified climate-related information needs that are considered by WGFD staff to be useful to future decision making. An understanding of what scientific knowledge is considered by decision-makers to be “salient” (directly relevant to decisions) is seen as a critical component to advancing science that is actually used (Cash et al. 2003). For this reason, co-production approaches to knowledge generation emphasize the importance of deriving research questions from end users rather than from researchers alone (Mach et al. 2020) and of having those questions be decision-driven (Vincent et al. 2018). The case study presented here offers specific methods that embrace those principles and can be used to elicit from managers what types of climate-related information would be useful to their decisions. These methods included a combination of discussions at a participatory workshop that generated a list of climate information needs with a follow-up survey that asked about the usefulness of each of those needs to agency managers and how the information might be used. The workshop session grounded the discussion of climate information needs in the context of specific management challenges and decisions being made by the focal agency, and the survey refined what was initially a long list of needs to a smaller number that were deemed especially useful to participating managers.

The resulting list of high-priority climate information needs can help the agency direct limited time and attention to addressing those needs that would be most directly relevant to management decisions in the coming years. It can also help the agency determine which information gaps can be addressed or filled directly by the agency, when they have the relevant expertise and capacity, versus others that might be well-suited for collaborations with outside researchers. These salient information needs can also inform the work of scientists interested in advancing actionable climate science. It is as yet unknown if this co-production effort will lead to research projects that address the identified climate change information needs.

### Recommendations, Limitations, and Potential Future Research Stemming from the Case Study

Overall, the agency core team indicated a high level of satisfaction with the process and felt that our methods could be useful for other natural resource management agencies looking to integrate climate change into planning and decision-making. Drawing on results from interviews, surveys, and our collective reflections on the project, we offer several recommendations related to the timing of co-production activities relative to the planning or decision-making process, allowing ample time for sharing and translating technical information, creating opportunities for a diversity of participants to contribute knowledge and perspectives, and employing a range of approaches to enable those contributions (Table 2).

**Table 2:** Recommendations stemming from this case study for integrating climate change into natural resource management planning and decision making

Recommendation	Details
Time the climate change co-production activities to align with the management planning or decision-making process.	Timing co-production efforts to align with management decision-making processes is a critical component to generating actionable information. In this effort, climate change workshops and other activities were designed to coincide with the SHP update timeline; however, co-production activities could have started even earlier in the planning process to allow for more time for gathering, sharing, and discussing climate change information.
Allow ample time for sharing and digesting technical information, and discussing implications for management.	Technical data on climate change trends, projections, and impacts can take time for information users to process. For this project, we presented a series of climate change talks in a roughly 2-hour block at the start of the participatory workshop, and shared with participants summaries of relevant climate projections for discussion during workshop breakout sessions. Delivering that same information in a series of shorter sessions or webinars spread out over a few weeks might have allowed for better assimilation of information and even more productive discussions at the workshop.
Create opportunities for participants with a range of perspectives to be engaged in the process.	The project, along with concurrent activities related to the SHP update led by the agency core team, engaged a substantial number of agency staff representing a range of positions from local biologists and field-based managers to supervisory managers and agency leaders. This likely increased buy-in for the final product and the ways that climate change was incorporated. We recommend employing multiple techniques for allowing such participation and contributions, such as workshops and surveys, and taking advantage of both virtual and in-person opportunities for engagement (see below). Although we were unable to hold repeated workshop sessions, doing so could allow for even greater participation by accommodating more peoples’ schedules and availability.
Employ a mix of virtual and in-person techniques.	Virtual meetings or workshops can allow for the inclusion of more outside researchers, improved representation by individuals who might not be able to travel to an in-person meeting, and the ability to use varied modes for contributing ideas (e.g., on-line documents that can be edited in real time by participants). However, these virtual approaches cannot fully replicate the benefits of building relationships through in-person interactions, especially the more nebulous yet important informal discussions that take place during coffee breaks or over meals. Finding creative ways to combine these approaches might offer the “best of both worlds” while also addressing some of the challenges mentioned above related to spreading out sessions over time and increasing the level of engagement and participation.

An important consideration when employing a co-production process like the one presented here is that the knowledge and products that are produced are inevitably shaped by those managers, researchers, and boundary spanners that are invited and choose to participate. The outcomes of this case study might have looked different if different individuals—with different knowledge systems, biases, communications abilities, etc.—had participated in the co-production process. This argues for involving as many individuals and knowledge systems as possible, in addition to having a transparent process and well-documented products that allow others to examine, and potentially critique the outputs.

Given that information use can lag behind the implementation of co-production activities, future work could continue to track how managers at WGFD use climate adaptation information produced during this case study. For example, projects that receive funding using the new scoring criteria described in the 2020 SHP could be analyzed for whether they are more likely to address climate change compared to projects that were funded before the 2020 SHP update was released. Interviews with WGFD staff that submit proposals for annual funding could also examine whether and to what extent the incorporation of climate change into the 2020 SHP and the new scoring criteria influenced the design of proposed projects.

## Conclusion

With this case study, we aimed to go beyond general principles for knowledge co-production to describe specific methods and evaluate their effectiveness. The methods that we trialed were considered by participating managers to be effective at co-producing usable climate change information and relevant to others looking to consider climate change in natural resource management planning and actions. Examples of information use documented during the nearly 1-year project included the incorporation of co-produced climate-informed strategies into an updated management plan and an increase in participating managers’ knowledge about climate change, its impacts, and adaptation strategies relevant to their work. The process also identified a list of climate change information needs deemed salient to managers’ decisions, which can be used to drive future actionable science research. Ultimately, the case study offers practical, effective, and replicable co-production methods that are relevant to managers and scientists in the United States and beyond that are looking to conduct knowledge co-production efforts with similar goals and desired outcomes.

## Supplementary information

Supplementary Materials

## Data Availability

Data generated by this study is available at the USGS Science Base catalog: https://www.sciencebase.gov/catalog/item/5b33b928e4b040769c172ee0.

## References

[CR1] Archie KM, Dilling L, Milford JB, Pampel FC (2012). Climate change and western public lands. Ecol Soc.

[CR2] Archie KM, Dilling L, Milford JB, Pampel FC (2014). Unpacking the ‘information barrier’: comparing perspectives on information as a barrier to climate change adaptation in the interior mountain West. J Environ Manag.

[CR3] Bamzai-Dodson A, Cravens AE, Wade AA, McPherson RA (2021). Engaging with stakeholders to produce actionable science: a framework and guidance. Weather, Clim, Soc.

[CR4] Barsugli J, Guentchev G, Horton R (2013). The practitioner’s dilemma: How to assess the credibility of downscaled climate projections. Eos, Trans Am Geophys Union.

[CR5] Bednarek AT, Shouse B, Hudson CG, Goldburg R (2016). Science-policy intermediaries from a practitioner’s perspective: the Lenfest Ocean Program experience. Sci Public Policy.

[CR6] Bednarek AT, Wyborn C, Cvitanovic C (2018). Boundary spanning at the science–policy interface: the practitioners’ perspectives. Sustain Sci.

[CR7] Beier P, Hansen L, Helbrecht L, Behar D (2017). A how-to guide for coproduction of actionable science. Conserv Lett.

[CR8] Bell S, Shaw B, Boaz A (2011). Real-world approaches to assessing the impact of environmental research on policy. Res Eval.

[CR9] Briley L, Brown D, Kalafatis SE (2015). Overcoming barriers during the co-production of climate information for decision-making. Clim Risk Manag.

[CR10] Carter S, Steynor A, Vincent K et al. (2019) Co-production of African weather and climate services, Manual. Future climate for Africa and Weather and climate information services for Africa, Cape Town, https://futureclimateafrica.org/coproduction-manual. Accessed 17 July 2022

[CR11] Cash DW, Borck JC, Patt AG (2006). Countering the loading-dock approach to linking science and decision making: comparative analysis of El Niño/Southern Oscillation (ENSO) forecasting systems. Sci, Technol, Hum Values.

[CR12] Cash DW, Clark WC, Alcock F (2003). Knowledge systems for sustainable development. Proc Natl Acad Sci.

[CR13] Cook C, Mascia M, Schwartz M (2013). Achieving conservation science that bridges the knowledge–action boundary. Conserv Biol.

[CR14] Crausbay S, Cross M (2019) State agency priorities for decisions that may be affected by climate variability or change: results from interviews with state fish and wildlife managers in the North Central region. Report for the North Central Climate Adaptation Science Center. https://www.sciencebase.gov/catalog/item/5d498835e4b01d82ce8de569. Accessed 14 July 2022

[CR15] Cross M, Dey P, Tator I, et al. (2020) Climate change & management of river, riparian, and wetlands habitats in Wyoming: summary from Wyoming Game and Fish Department (WGFD) climate change workshop. Wildlife Conservation Society & WGFD. https://wgfd.wyo.gov/WGFD/media/content/PDF/Habitat/2020-WGFD-WCS-Workshop-Report.pdf. Accessed 20 July 2022

[CR16] Cross MS, Zavaleta ES, Bachelet D (2012). The Adaptation for Conservation Targets (ACT) framework: a tool for incorporating climate change into natural resource management. Environ Manag.

[CR17] Dilling L, Lemos MC (2011). Creating usable science: opportunities and constraints for climate knowledge use and their implications for science policy. Glob Environ Change.

[CR18] Djenontin INS, Meadow AM (2018). The art of co-production of knowledge in environmental sciences and management: lessons from international practice. Environ Manag.

[CR19] Goodrich KA, Sjostrom KD, Vaughan C (2020). Who are boundary spanners and how can we support them in making knowledge more actionable in sustainability fields. Curr Opin Environ Sustain.

[CR20] Hyman AA, Courtney S, McNeal KS, et al. (2022) Distinct pathways to stakeholder use versus academic contribution in climate adaptation research. Conserv Lett 10.1111/conl.12892

[CR21] IPCC (2022) Climate change 2022: impacts, adaptation, and vulnerability. In: Contribution of Working Group II to the Sixth Assessment Report of the Intergovernmental Panel on Climate Change [H.-O. Pörtner, D.C. Roberts, M. Tignor, E.S. Poloczanska, K. Mintenbeck, A. Alegría, M. Craig, S. Langsdorf, S. Löschke, V. Möller, A. Okem, B. Rama (eds)]. Cambridge University Press, UK

[CR22] Jagannathan K, Jones AD, Ray I (2021). The making of a metric: co-producing decision-relevant climate science. Bull Am Meteorol Soc.

[CR23] Jesiek B, Mazzurco A, Buswell N, Thompson J (2018). Boundary spanning and engineering: a qualitative systematic review. J Eng Educ.

[CR24] Kemp KB, Blades JJ, Klos PZ (2015). Managing for climate change on federal lands of the western United States: perceived usefulness of climate science, effectiveness of adaptation strategies, and barriers to implementation. Ecol Soc.

[CR25] Kolstad EW, Sofienlund ON, Kvamsås H (2019). Trials, errors, and improvements in coproduction of climate services. Bull Am Meteorol Soc.

[CR26] Lemos MC, Arnott JC, Ardoin NM (2018). To co-produce or not to co-produce. Nat Sustain.

[CR27] Lemos MC, Kirchhoff CJ, Ramprasad V (2012). Narrowing the climate information usability gap. Nat Clim Change.

[CR28] Mach KJ, Lemos MC, Meadow AM (2020). Actionable knowledge and the art of engagement. Curr Opin Environ Sustain.

[CR29] Meadow AM, Ferguson DB, Guido Z (2015). Moving toward the deliberate coproduction of climate science knowledge. Weather, Clim, Soc.

[CR30] Moser SC, Ekstrom JA (2010) A framework to diagnose barriers to climate change adaptation. Proc Natl Acad Sci 107:22026–22031. 10.1073/pnas.1007887107 10.1073/pnas.1007887107 PMC3009757 21135232

[CR31] Nel J, Roux D, Driver A (2016). Knowledge co-production and boundary work to promote implementation of conservation plans. Conserv Biol.

[CR32] Norström AV, Cvitanovic C, Löf MF (2020). Principles for knowledge co-production in sustainability research. Nat Sustain.

[CR33] Patton M (2002). Qualitative research and evaluation methods.

[CR34] Pelz D, Yinger JM, Cutler SJ (1978). Some expanded perspectives on use of social science in public policy. Major social issues: a multidisciplinary view.

[CR35] Posner SM, Cvitanovic C (2019). Evaluating the impacts of boundary-spanning activities at the interface of environmental science and policy: a review of progress and future research needs. Environ Sci Policy.

[CR36] Safford HD, Sawyer SC, Kocher SD (2017). Linking knowledge to action: the role of boundary spanners in translating ecology. Front Ecol Environ.

[CR37] Stein BA, Glick P, Edelson NA, Staudt A (2014). Climate-smart conservation: putting adaptation principles into practice.

[CR38] Tengö M, Hill R, Malmer P (2017). Weaving knowledge systems in IPBES, CBD and beyond—lessons learned for sustainability. Curr Opin Environ Sustain.

[CR39] VanderMolen K, Meadow AM, Horangic A, Wall TU (2020). Typologizing stakeholder information use to better understand the impacts of collaborative climate science. Environ Manag.

[CR40] Vincent K, Daly M, Scannell C, Leathes B (2018). What can climate services learn from theory and practice of co-production. Clim Serv.

[CR41] Wall TU, Meadow AM, Horganic A (2017). Developing evaluation indicators to improve the process of coproducing usable climate science. Weather, Clim, Soc.

[CR42] WGFD (2020) Statewide Habitat Plan 2020. Wyoming Game and Fish Department. https://wgfd.wyo.gov/getmedia/8ba62756-6d1c-4257-8644-82383dfa605a/SHP2020_Final. Accessed 14 July 2022

[CR43] Williams P (2002). The competent boundary spanner. Public Adm.

