# Supplementary material for: Tackling the Science Usability Gap in a Warming World: Co-Producing Useable Climate Information for Natural Resource Management
Source: Environ Manage. 2022 Sep 26;70(6):881–95. doi: 10.1007/s00267-022-01718-4 (PMC9510579; doi:10.1007/s00267-022-01718-4)
Supplement: Supplementary file 1 — Supplementary Materials [file 267_2022_1718_MOESM1_ESM.pdf]

Supplementary Materials

Title: Tackling the Science Usability Gap in a Warming World: Co-Producing Useable Climate Information for Natural Resource Management

Journal: Environmental Management

Authors: Molly S. Cross[1,*], Lauren E. Oakes[1,2] Heidi E. Kretser[1,3], Raymond Bredehoft[4], Paul Dey[4], Anika Mahoney[4], Noelle Smith[4], Ian Tator[4], Jim Wasseen[4]

1. Wildlife Conservation Society, Bronx, New York, USA
2. Stanford University, Department of Earth System Science, Stanford, California, USA
3. Cornell University, Department of Natural Resources and the Environment, Ithaca, New York, USA
4. Wyoming Game and Fish Department, Cheyenne, Wyoming, USA

* Corresponding author (mcross@wcs.org)

TABLE OF CONTENTS **Page**

Supplementary Materials 1: Methods for Step 1 (Identify Actors & Build Partnerships) 1
1.1 Co-Production Process Consent Form 1
1.2 Shared Goals Semi-Structured Interview Protocol 2
Supplementary Materials 2: Methods for Step 2 (Co-Explore Need) 3
2.1 Focus Group Discussion Guide 3
Supplementary Materials 3: Methods for Step 3 (Co-Develop Solution) 4
3.1 Information Needs Survey Protocol 4
Supplementary Materials 4: Methods and Detailed Results for Step 5 (Evaluation) 9
4.1 Pre-Workshop Survey Protocol 9
4.2 Post-Workshop Survey Protocol 11
4.3 Pre/Post Workshop Survey Analyses & Results 13
4.4 Post-Project Semi-Structured Interview Protocol 18

Supplementary Materials 1: Methods for Step 1 (Identify Actors & Build Partnerships)

1.1 Co-Production Process Consent Form

At the start of the project, the agency core team members reviewed and signed a consent form that covered the entire co-production process. The consent form included the following information:

Project: The Wildlife Conservation Society will work with the Wyoming Fish and Game Department to support decision-making for resource managers and scientists confronting climate change in the ecosystems they study and manage. Together, we will develop, apply, and evaluate a step-by-step co-production process for integrating climate science into the 2020 revision of the Wyoming Statewide Habitat Plan.

Sponsor: This project is funded by the North Central Climate Adaptation Science Center (NCCASC).

Time Involvement: Your participation in this project is voluntary. Throughout the project we will use mixed methods (e.g., semi-structured interviews, focus groups, participatory workshops, and surveys) that will be further developed, applied, and evaluated through project work with the Wyoming Fish and Game Department.

With your permission, we will keep paper and digital notes during all phases of this research. Some discussions may be recorded, and we may also use information from emails and other forms of communication. As the project evolves, you may be asked to complete one or several short surveys for evaluation purposes.

Project Outcomes: The results of this project will be published in journal articles and reports as well as presented at conferences. You may obtain copies of the results upon its completion by contacting the investigators listed above. Given this research is a co-production project by design, you may also have the opportunity to contribute to any publication(s) as a co-author, if this is a role that interests you.

Confidentiality: Your identity will be kept anonymous in any summaries of this work. Given that the case study will focus on management decisions by a public agency, it may be possible for readers to infer your identity based on your specific role within the agency. We expect that all agency staff and external partners involved will know the names of other participants.

Potential Risks and Benefits: Your participation in this study will involve minimal risk. Intentionally, we have chosen a collaborative and voluntary approach to this research. Given that the study focuses on the management of a public resource, people may be able to associate certain statements to you specifically despite our efforts to maintain confidentiality. Direct benefits to participants could include assistance in the synthesis and application of relevant best available science to the 2020 revision of the Wyoming Strategic Habitat Plan.

Contact for information about the study: If you have any questions, concerns or require more information about the study, you may contact Dr. Molly Cross (mcross@wcs.org or Dr. Lauren Oakes (loakes@wcs.org).

Contact for concerns or complaints: If you have any concerns or complaints about your rights as a research participant and/or your experiences while participating in this co-production process, contact wcsirb@wcs.org.

Consent: Your participation in this study, a co-production process, is voluntary and you have the right to refuse to participate. If you decide to take part, you may choose to stop participating at any time during any phase of the co-production process without giving a reason and without any negative consequences. Withdrawal from the study after participating in any phase(s) can be arranged by contacting Dr. Molly Cross at the contact information provided.

Please check yes or no to agree or disagree with the following statements.

> I have received a copy of this consent form for my own records. Yes ____ No ____.
>
> I accept that my participation in any phase or phases of this co-production research process will be recorded as data in the form of digital and/or paper notes, and/or responses to online survey(s): Yes ____ No ____.
>
> I also accept that my participation may be recorded for transcription in a group or one-on-one setting: Yes ____ No ____.

Your signature indicates that you consent to participate in this study.

1.2 Shared Goals Semi-Structured Interview Protocol

Interview Recruitment: As described in the Case Study Selection section of the main manuscript, the boundary actor interviewed each member of the core agency team to solicit their input into the shared goals for the co-

production process. The boundary actor explained the purpose of the semi-structured interviews and invited the agency core team members to participate on a phone call at the start of the project.

Interview Script:

- First, would you like me to go over this study's goals, or answer any questions you might have about the project? The purpose of this project is to advance collaborative methods for enabling management decisions that anticipate climate variability and change. This project is a form of participatory action research (PAR); together, we will develop and evaluate an effort to "co-produce" science synthesis and planning that is tied to a specific management decision - the 2020 revision of the Wyoming SHP. We will then share the results and lessons learned from this project through presentations and a peer-reviewed publication.

- This project has been approved by the Institutional Review Board of the Wildlife Conservation Society, and we treat this interview as confidential.

- As you recall from the consent form I'm going to be recording today's call to help with notetaking and accuracy in documenting your responses, so I'm going to start the recording now.

Question 1. What benefits are you hoping to gain from being involved in this co-production project, focused on the 2020 SHP revision? *(Explore in terms of current knowledge, available information, relationship-building, possible management-decision outcomes.)*

Question 2. How would you describe success for our work together over the coming year, or perhaps longer?

Question 3. What deliverables are you hoping to gain from our work together?

Question 4. When during the timeline of the SHP revision would information from our project be most useful?

Question 5. Can you point me toward any scientists whose research may be relevant to the decision opportunity that you've identified?

Supplementary Materials 2: Methods for Step 2 (Co-Explore Need)

2.1 Focus Group Discussion Guide

Focus Group Recruitment: Invitees for the Focus Group included the agency core team and 12 climate experts from outside of the agency. As described in the Co-Production Process - Methods & Results section of the main manuscript, the boundary actor identified invited climate experts through a scan of scientific literature (i.e., peer-reviewed studies and agency science reports), interviews with the agency core team (described in S1.2), and informal conversations with climate change researchers in the region. The boundary actor talked with each of the climate experts by phone to personally invite them to participate in a Focus Group meeting to discuss the planning and decision-making context to be informed by the co-production process.

Focus Group Script:

Thank you for taking the time to attend this meeting, which is not only a key part of preparing for our upcoming workshop, but also a critical step in efforts to "co-produce" and apply useable climate science for natural resource decision making. In this "co-production" project we are focused on starting with the decision context, in this case the upcoming revision of Wyoming's Statewide Habitat Plan (or SHP). Therefore, we wanted to bring together the core team working on the SHP revision from WGFD with the non-WGFD climate experts that we have invited to participate in the workshop, so that WGFD staff can provide the climate experts with some background information

on the SHP and the types of priorities that it includes and decisions that it supports. This background will hopefully help our non-WGFD partners prepare for the workshop and think ahead to data and resources that will be relevant. Formally through presentations, but also informally through participating in the workshop discussions.

Before we begin, I wanted to remind everyone that we are also engaged in a research project that is studying our collaborative process so that we can share what we do and learn from this project with others that are interested in methods and approaches to co-producing climate science information that is relevant and used in management decisions. Therefore, what we discuss during this meeting may be shared at presentations or in peer-reviewed journals about how the collaboration has worked (or not). As explained in the consent forms that you have all received and signed, we will not associate your name with your comments. We ask all attendees to similarly respect that confidentiality, although we recognize that we have no control over what each individual does with the information shared here today. As we also explained in the consent forms, I am going to record today's meeting for ease and accuracy in notetaking.

By participating in this call, I assume you are giving your consent to the recording.

With that, I'm going to hit to record button and get started

[begin recording]

<u>Focus Group Agenda/Discussion Guide:</u>

- Opening script (above) and Introductions (10 minutes)
- WGFD staff provide an overview of the Statewide Habitat Plan – what kinds of priorities it includes, what types of decisions it supports, what the timeline and workplan is for the 2020 revision, how this climate change piece fits in, and what habitat types are we focused on for the April 28-30 Workshop (10 minutes)
- Discussion prompts:
    - Theme 1 - How have SHP priorities been identified in the past, and what is the process for revising the SHP in 2020? (20 mins)
        - What aspects of the SHP priorities are flexible? What aspects are limited? (e.g., could the agency decide to modify the boundaries of priority areas? Adjust what action are needed within priority areas? Etc.)
        - What kinds of science information (analyses, data, research results, expert opinion, etc.) has previously been used to inform the decision? What is planned for the 2020 revision?
        - How does the SHP deal with risks? Uncertainty?
    - Theme 2 – What climate issues are of particular concern going into the workshop? (15 mins)
        - Are there particular aspects of the SHP that might allow for a consideration of climate change and its impacts? (e.g., selection of priority places? Identification of priority actions?)
        - What concerns do you have about climate change in terms of how it might affect SHP goals and priorities?
- Wrap up – Brief workshop overview; Thank everyone for their time

---

Supplementary Materials 3: Methods for Step 3 (Co-Develop Solution)

3.1 Information Needs Survey Protocol

<u>Introductory text:</u>
What does the Agency need to know in order to make better decisions in the next 5 years?
During a workshop held April 28-30, Wyoming Game and Fish Department (WGFD) staff and external partners identified a wide range of information needs (e.g., research, analyses, products, inventories, etc.) that would help the Agency make better climate-informed management decisions about river, riparian, and wetland habitats in the next 5 years. Your responses on this survey will help WGFD prioritize these information needs by identifying ones that are

considered by WGFD staff to be the most useful to informing management projects and decisions related to river, riparian, and wetland habitats. Even if you did not participate in the workshop, we are interested in your responses about which of the identified information needs are most useful to your work.

This survey should take approximately 20 Minutes to complete.

The survey is also part of a research study led by the Wildlife Conservation Society to evaluate methods for incorporating climate science into natural resource management decisions. Results from this survey may be shared via peer-reviewed publications and presentations. Your participation in this survey is voluntary, and you have the right to refuse to participate. All information received will remain anonymous, and we anticipate minimal risk from your participation.

If you have any concerns or complaints about your rights as a participant and/or your experiences while participating in this survey, please contact wcsirb@wcs.org (reference project #20-04). Please do not hesitate to contact Molly Cross (mcross@wcs.org) if you have any questions regarding this research or how the results will be used.

Survey protocol:

Question 1. Please indicate the primary focus of your work (check all that apply):

- Aquatic habitat
- Fisheries
- Wildlife
- Terrestrial habitat
- Wildlife Habitat Management Areas
- Wetlands
- Department Leadership Staff
- Other ___{fill in}____

Question 2. Did you attend the April 28-30, 2020 WGFD Climate Change Workshop? Yes/No

If yes, please indication which sessions you attended (check all that apply)

- ○ Climate Science Presentations (Tuesday afternoon)
- ○ Climate Change Impacts (Wednesday morning)
- ○ Assessing Relative Climate Change Vulnerability (Wednesday afternoon)
- ○ What’s Different About Climate-Informed Management (Thursday morning)
- ○ Priority Climate-Informed Management Strategies for the SHP (Thursday afternoon)
- ○ Identifying Research and Information Needs (Thursday afternoon)

In Questions 3-13, you will be asked to indicate how useful each of the information needs identified at the workshop are to your ability to consider the effects of climate change in your work on river, riparian, or wetland ecosystems. Because workshop discussions generated a large number of information needs (e.g., research, analyses, products, inventories), we have divided them into smaller groups organized by themes to make it easier to answer the survey questions. Later in the survey you will have an opportunity to provide additional details about those information needs that you consider to be most useful to your work. You will also have a chance to add any climate change information needs not listed in the survey that you consider to be very useful to your work.

Question 3. Please indicate how useful each of the following information needs related to Riparian & Wetland Ecosystems are to your ability to consider the effects of climate change in your work on river, riparian, or wetland habitats. [Options: Not At All Useful, Slightly Useful, Moderately Useful, Useful, Very Useful]

- Determine whether there is spatial variation across Wyoming in woody vs. herbaceous plant responses to changes in climate (e.g., temperature, evapotranspiration, drought).

- Determine whether there are likely to be significant changes in synchrony related to cottonwood germination and growth success.
- Conduct habitat assessments to determine riparian resilience and appropriate diversity of habitats within the system (could include incorporating climate vulnerability into other existing habitat assessment protocols and methods).
- Investigate how different amounts of change in precipitation and/or temperature would lead to changes in a resource of interest (e.g., wetland area fluctuations with respect to changes in precipitation).

Question 4. Please indicate how useful each of the following information needs related to Invasive Species are to your ability to consider the effects of climate change in your work on river, riparian, or wetland habitats. [Options: Not At All Useful, Slightly Useful, Moderately Useful, Useful, Very Useful]

- Determine which invasive species we might expect to see that are not yet in Wyoming.
- Determine whether there are likely to be significant changes in synchrony favoring Russian olive and salt cedar.
- Investigate relationships between invasive plants and invasive fish species to understand potential management actions.
- Analyze the existing and potential future location of barriers in key watersheds relative to keeping native and non-native fish species apart.
- Identify management or habitat actions that disadvantage invasive fish and plant species.

Question 5. Please indicate how useful each of the following information needs related to Climate Change Vulnerability Assessments are to your ability to consider the effects of climate change in your work on river, riparian, or wetland habitats. [Options: Not At All Useful, Slightly Useful, Moderately Useful, Useful, Very Useful]

- Conduct species-specific climate change vulnerability assessments at local scale (e.g., within wildlife habitat management areas or sub-watersheds).
- Conduct species-specific climate change vulnerability assessments at regional scale (e.g., large watersheds).
- Conduct species-specific climate change vulnerability assessments at the statewide scale (e.g., assess the relative climate change vulnerability of all WHMAs or sub-watersheds across the state).
- Develop database of species-specific tolerances to changes in climate (aka climate vulnerability).

Question 6. Please indicate how useful each of the following information needs related to Climate Refugia, Prioritization, and Planning are to your ability to consider the effects of climate change in your work on river, riparian, or wetland habitats. [Options: Not At All Useful, Slightly Useful, Moderately Useful, Useful, Very Useful]

- Identify climate refugia (within and outside of historic range) for imperiled species that may serve as key source populations and allow habitat limitations to be addressed.
- Identify potential translocation sites for species of conservation concern that consider future climate conditions not just current climate conditions.
- Develop a standardized, systematic protocol for evaluating and prioritizing watersheds for protection and restoration as related to climate change, that considers aquatic and terrestrial needs.
- Analyze management objectives of Wildlife Habitat Management Areas (WHMAs) relative to climate change predictions.

Question 7. Please indicate how useful each of the following information needs related to Aquatic Habitat and Fisheries are to your ability to consider the effects of climate change in your work on river, riparian, or wetland habitats. [Options: Not At All Useful, Slightly Useful, Moderately Useful, Useful, Very Useful]

- Conduct site-specific studies of base flows needed to allow fish survival during periods of high water temperatures.
- Determine thermal limits for specific species.
- Develop an inventory of water temperatures by watershed and prioritize management based on species-specific tolerances.
- Develop fish habitat models that incorporate climate variables into stream suitability/vulnerability analyses for species and assemblages; Identify streams that could become suitable for particular species or assemblages under future climate scenarios.
- Conduct a range-wide genetic assessment of Yellowstone Cutthroat Trout to determine genetic variation of populations to guide future protection and management actions.
- Determine whether there are likely to be significant changes in synchrony between native cutthroat trout spawning and changing water temperatures and runoff timing.

Question 8. Please indicate how useful each of the following information needs related to Fish Passage and Stream Connectivity are to your ability to consider the effects of climate change in your work on river, riparian, or wetland habitats. [Options: Not At All Useful, Slightly Useful, Moderately Useful, Useful, Very Useful]

- Develop or adjust design criteria for fish passage structures and culverts that account for larger floods and lower base flows.
- Develop a statewide climate-informed stream connectivity assessment.
- Project future instream habitat conditions (to prioritize fish passage projects).
- Develop an inventory of natural fish barriers.

Question 9. Please indicate how useful each of the following information needs related to Hydrology & Water Balance are to your ability to consider the effects of climate change in your work on river, riparian, or wetland habitats. [Options: Not At All Useful, Slightly Useful, Moderately Useful, Useful, Very Useful]

<u>Hydrology/Water Balance</u>

- Determine water holding capacity in shallow alluvial riparian areas, as a function of different valley forms, geology, land use, and vegetation characteristics.
- Understand how upland habitat treatments (juniper removal, sagebrush mowing, etc.) link to water release into the watershed and system impacts with more intense precipitation events.
- Investigate resiliency and impacts in different hydrologic provinces: e.g., Snowmelt prairie streams vs. non-snowmelt prairie streams.

Question 10. Please indicate how useful each of the following information needs related to Stream Restoration are to your ability to consider the effects of climate change in your work on river, riparian, or wetland habitats. [Options: Not At All Useful, Slightly Useful, Moderately Useful, Useful, Very Useful]

- Identify places with higher future risk of flooding to prioritize floodplain reconnection with stream restoration to reduce impacts.
- Predict future bankfull discharges and sediment transport resulting from increased peak flows and precipitation intensity, for use in stream restoration design.
- Develop prairie stream Best Management Practices (BMPs) for habitat enhancement given predicted climate changes.
- Collect reference reach information at existing functioning prairie stream sites to provide a template for restoration.

Question 11. Please indicate how useful each of the following information needs related to Beaver and Other Process-Based Restoration Approaches are to your ability to consider the effects of climate change in your work on river, riparian, or wetland habitats. [Options: Not At All Useful, Slightly Useful, Moderately Useful, Useful, Very Useful]

- Determine how beaver dam analogs, beaver, Zeedyk structures, etc. affect the timing and quantity of water delivered to downstream water rights holders.
- Determine how beaver dam analogs, beaver, Zeedyk structures, etc. affect shallow alluvial aquifers and riparian areas.
- Develop an up-to-date and accurate BRAT (Beaver Restoration Assessment Tool) model.
- Assess beaver translocation success or failure to determine what drives survival and establishment of colonies and understand spatial variability.

Question 12. Please indicate how useful each of the following information needs related to Baseline Data and Monitoring are to your ability to consider the effects of climate change in your work on river, riparian, or wetland habitats. [Options: Not At All Useful, Slightly Useful, Moderately Useful, Useful, Very Useful]

- Develop novel methods for conducting large scale monitoring efficiently (remote sensing, drones, loggers) and consider less monitoring in some cases.
- Increase streamflow and wetlands monitoring to build on historic monitoring and track changes in water quantities, timing and use.
- Develop statewide stream and riparian condition information depicting departure from expected conditions stratified by valley type, slope, and physiographic province; to indicate where streams are most degraded /furthest from functioning.

Question 13. Please indicate how useful each of the following information needs related to Water Management are to your ability to consider the effects of climate change in your work on river, riparian, or wetland habitats. [Options: Not At All Useful, Slightly Useful, Moderately Useful, Useful, Very Useful]

- Explore the feasibility of capturing water runoff from irrigation and recirculating for further use.
- Develop a better understanding and examples of tradeoffs for water use and wildlife benefits for flood versus pivot irrigation.
- Analyze tradeoffs between managing water use for instream habitats and out-of-stream habitats (e.g., wetlands) (i.e., determine habitat and ecosystem function gains and losses per cfs).

Question 14. For those research and information needs that you ranked as "Very Useful", please provide any relevant details about the research or information needed (e.g., for which species, which geographies, at what scale, etc.). [text box response]

Question 15. For those research and information needs that you ranked as "Very Useful", please comment on how you would use the information or research results in your work. [text box response]

Question 16. If there are any information needs (e.g., research, analyses, products, inventories, etc.) that have not already been mentioned that you feel would be "Very Useful" to considering climate change in your work, please provide details. [text box response]

Question 17. OPTIONAL - If you are also interested in participating in follow up discussions regarding those information needs that you have identified as being useful to your work, please enter your email address. [text box response]

Supplementary Materials 4: Methods and Detailed Results for Step 5 (Evaluation)

4.1 Pre-Workshop Survey Protocol

Introductory text:

Thank you for taking the time to complete this survey in advance of the April 28-30, 2020 climate change workshop being hosted by the Wyoming Game and Fish Department (WGFD) and the Wildlife Conservation Society. This pre-workshop survey will help us evaluate the effectiveness of the workshop at increasing WGFD staff's knowledge about climate change and its impacts, comfort with considering climate change in natural resource management decisions, and interactions with climate experts that are external to WGFD. **The survey should only take 5-10 minutes of your time.**

The survey is also part of a research study being conducted by the Wildlife Conservation Society to evaluate methods for incorporating climate science into natural resource management decisions. Results from this survey may be shared via publications and presentations. Your participation in this survey is voluntary, and you have the right to refuse to participate. All information received will remain anonymous, and we anticipate minimal risk from your participation.

If you have any concerns or complaints about your rights as a participant and/or your experiences while participating in this survey, please contact (wcsirb@wcs.org; reference project #20-04).

Please do not hesitate to contact Molly Cross (mcross@wcs.org) if you have any questions regarding this research project or how the results will be used.

Survey Protocol:

1. **How knowledgeable are you about climate change projections and impacts as they relate to your work?** Sliding Scale from 0-10 with 0 = Not at all Knowledgeable; 5 = Neutral; 10 = Very Knowledgeable

2. **How comfortable are you with integrating climate change information into your work?** Sliding Scale from 0-10 with 0 = Not at all Comfortable; 5 = Neutral; 10 = Very Comfortable

3. **How familiar are you with approaches and tools for climate change-informed conservation planning?** Sliding Scale from 0-10 with 0 = Not at all Familiar; 5 = Neutral; 10 = Very Familiar

4. **How familiar are you with climate change adaptation strategies or actions that are relevant to your work?** Sliding Scale from 0-10 with 0 = Not at all Familiar; 5 = Neutral; 10 = Very Familiar

5. **Please indicate how much you agree with the following statement: I have the knowledge I need to do my job in the face of climate change.** Sliding Scale from 0-10 with 0 = Strongly Disagree; 5 = Not Sure; 10 = Strongly Agree

6. **Please indicate how much you agree with the following statement: I have the tools I need to do my job in the face of climate change.** Sliding Scale from 0-10 with 0 = Strongly Disagree; 5 = Not Sure; 10 = Strongly Agree

7. **Do you anticipate needing to do anything differently in your job because of a changing climate?** Sliding Scale from 0-10 with 0 = Definitely Not; 5 = Not Sure; 10 = Definitely Yes

8. **In the past 6 months, where have you obtained information (data, reports, research results) that improves your understanding of climate change and its impacts? (CHECK ALL THAT APPLY)**

______Federal agencies
______Wyoming Game and Fish Department
______Other State agencies
______Conservation/wildlife non-governmental organizations
______Universities
______Association of Fish and Wildlife Agencies (AFWA) / Western Association of Fish and Wildlife Agencies (WAFWA)
______Conferences
______Workshops
______Discussions with experts
______Other

9. **In the past 6 months, please indicate how frequently you have directly interacted with individuals from the following groups to share information about climate change and its impacts.**

	Never	Very Rarely	Rarely	Occasionally	Frequently	Very Frequently
Federal agency scientists	0	1	2	3	4	5
State wildlife agency scientists	0	1	2	3	4	5
Other State agency scientists	0	1	2	3	4	5
Non-governmental organization (NGO) scientists	0	1	2	3	4	5
Academics/University scientists	0	1	2	3	4	5
Association of Fish and Wildlife Agencies (AFWA) / Western Association of Fish and Wildlife Agencies (WAFWA)	0	1	2	3	4	5
Other____________________	0	1	2	3	4	5

10. **How accessible are climate scientists to you personally for sharing information relevant to your work?**
Sliding Scale from 0-10 with 0 = Not at all Accessible; 5 = Neutral; 10 = Very Accessible

LAST QUESTION - P**lease enter the following 6-digit combination: The Month and Day of your birthday followed by the first two letters of your Mother's first name** (for example, if your birthday is January 15 and your mother's first name is Mary you would enter 0115MA). We will ask you to re-enter that same 6-digit/letter code into a POST-workshop survey that we will send out after the workshop. This will allow us to compare some of your pre-workshop and post-workshop responses in a way that keeps your survey responses anonymous. [Short Answer field]

4.2 Post-Workshop Survey Protocol

Introductory text:

Thank you for participating in the April 28-30, 2020 climate change workshop hosted by the Wyoming Game and Fish Department (WGFD) and the Wildlife Conservation Society. We are asking you to complete a POST-workshop survey that will help us evaluate the effectiveness of the workshop. Even if you did not respond to the PRE-workshop survey, your responses to this POST-workshop survey will be very informative.

The survey should only take 15 minutes to complete.

The survey is also part of a research study being conducted by the Wildlife Conservation Society to evaluate methods for incorporating climate science into natural resource management decisions. Results from this survey may be shared via peer-reviewed publications and presentations. Your participation in this survey is voluntary, and you have the right to refuse to participate. All information received will remain anonymous, and we anticipate minimal risk from your participation.

If you have any concerns or complaints about your rights as a participant and/or your experiences while participating in this survey, please contact wcsirb@wcs.org (reference project #20-04). Please do not hesitate to contact Molly Cross (mcross@wcs.org) if you have any questions regarding this research or how the results will be used.

Survey protocol:

1. Please enter the following 6-digit combination: The Month and Day of your birthday followed by the first two letters of your Mother's first name (for example, if your birthday is January 15 and your mother's first name is Mary you would enter 0115MA). If you participated in the PRE-workshop survey that we distributed before the April 28-30 workshop, you will recall that we asked you to enter this same 6-digit/letter code. Providing this code will allow us to compare your pre-workshop and post-workshop responses in a way that keeps your survey responses anonymous. Even if you did not complete the pre-workshop survey, your responses on this post-workshop survey will be very informative.

2. Which sessions of the workshop did you attend? (check all that apply)

Climate Science Presentations (Tuesday 1:00pm-3:15pm)
Climate Change Impacts (Wednesday 10:00am-12:00pm)
Assessing Relative Climate Change Vulnerability (Wednesday 1:00-3:30pm)
What's Different About Climate-Informed Management (Thursday 10:00am-12:00pm)
Priority Climate-Informed Management Strategies for the SHP (Thursday 1:00-3:30pm)
Identifying Research and Information Needs (Thursday 1:00-3:30pm)

3. After participating in the workshop, how knowledgeable are you about climate change projections and impacts as they relate to your work? Sliding Scale from 0-10 with 0 = Not at all Knowledgeable; 5 = Neutral; 10 = Very Knowledgeable

4. As a result of the workshop, did you gain new knowledge about climate change projections and impacts?
Yes / No

5. After participating in the workshop, how comfortable are you with integrating climate change information into your work? Sliding Scale from 0-10 with 0 = Not at all Comfortable; 5 = Neutral; 10 =

Very Comfortable

6. As a result of the workshop, do you feel more comfortable integrating climate change information into your work? Yes / No

7. After participating in the workshop, how familiar are you with approaches and tools for climate change informed conservation planning? Sliding Scale from 0-10 with 0 = Not at all Familiar; 5 = Neutral; 10 = Very Familiar

8. As a result of the workshop, do you feel more familiar with approaches and tools for climate change informed conservation planning? Yes / No

9. After participating in the workshop, how familiar are you with climate change adaptation strategies or actions that are relevant to your work? Sliding Scale from 0-10 with 0 = Not at all Familiar; 5 = Neutral; 10 = Very Familiar

10. As a result of the workshop, do you feel more familiar with climate change adaptation strategies or actions that are relevant to your work? Yes / No

11. Please indicate how much you agree with the following statement: I have the knowledge I need to do my job in the face of climate change. Sliding Scale from 0-10 with 0 = Strongly Disagree; 5 = Not Sure; 10 = Strongly Agree

12. What knowledge do you <u>not</u> have that you feel is necessary to do your job in the face of climate change? [text box response]

13. Please indicate how much you agree with the following statement: I have the tools I need to do my job in the face of climate change. Sliding Scale from 0-10 with 0 = Strongly Disagree; 5 = Not Sure; 10 = Strongly Agree

14. What tools do you <u>not</u> have that you feel are necessary to do your job in the face of climate change? [text box response]

15. Do you anticipate needing to do anything differently in your job because of a changing climate? Sliding Scale from 0-10 with 0 = Definitely Not; 5 = Not Sure; 10 = Definitely Yes

16. If you anticipate needing to do something differently in your job (to some extent), please provide some examples. [text box response]

17. As a result of participating in this workshop, did you learn of new materials, tools, or resources that you can use to improve your understanding of climate changes and impacts? Yes / No; If yes, please provide some examples. [text box response]

18. As a result of participating in this workshop, did you "meet" any new individuals with whom you will likely develop or share information about climate science in the future? Yes / No; If Yes, please provide some examples. [text box response]

19. Please indicate how useful the following workshop sessions were <u>to your work</u>. Likert scale: Not at all useful; Somewhat Useful; Useful; Very Useful; Not applicable/Did not attend

Climate Science Presentations (Tuesday 1:00pm-3:15pm)
Climate Change Impacts (Wednesday 10:00am-12:00pm)
Assessing Relative Climate Change Vulnerability (Wednesday 1:00-3:30pm)
What's Different About Climate-Informed Management (Thursday 10:00am-12:00pm)
Priority Climate-Informed Management Strategies for the SHP (Thursday 1:00-3:30pm)
Identifying Research and Information Needs (Thursday 1:00-3:30pm)

20. Please indicate how useful the following workshop sessions were to <u>creating or strengthening your relationships with external climate experts</u>. Likert scale: Not at all useful; Somewhat Useful; Useful; Very Useful; Not applicable/Did not attend

Climate Science Presentations (Tuesday 1:00pm-3:15pm)
Climate Change Impacts (Wednesday 10:00am-12:00pm)
Assessing Relative Climate Change Vulnerability (Wednesday 1:00-3:30pm)
What's Different About Climate-Informed Management (Thursday 10:00am-12:00pm)
Priority Climate-Informed Management Strategies for the SHP (Thursday 1:00-3:30pm)
Identifying Research and Information Needs (Thursday 1:00-3:30pm)

21. Do you have any other comments about the workshop, including suggestions for improvement? [text box response]

22. What topics were <u>not</u> covered during the workshop that you wish could be covered in the future? *Even if you did not attend all the sessions, please comment based on your review of the agenda and the sessions you did attend.* [text box response]

23. Is there anything that you feel WGFD needs to do as a result of discussions at the workshop to improve how staff include climate change information in decision-making? [text box response]

4.3 Pre/Post Workshop Survey Analyses & Results

We analyzed several pre- and post-workshop survey responses related to the effectiveness of the workshop.

First, we present results from three binary, yes/no questions from the post-workshop survey regarding whether, as a result of the workshop, agency participants: 1) gained new knowledge about climate change projections and impacts, 2) felt more comfortable integrating climate change into their work, and 3) were more familiar with climate change adaptation strategies and actions relevant to their work. For all three of those questions, 89% (31 out of 35) responded "yes" (Figure S1).

a)

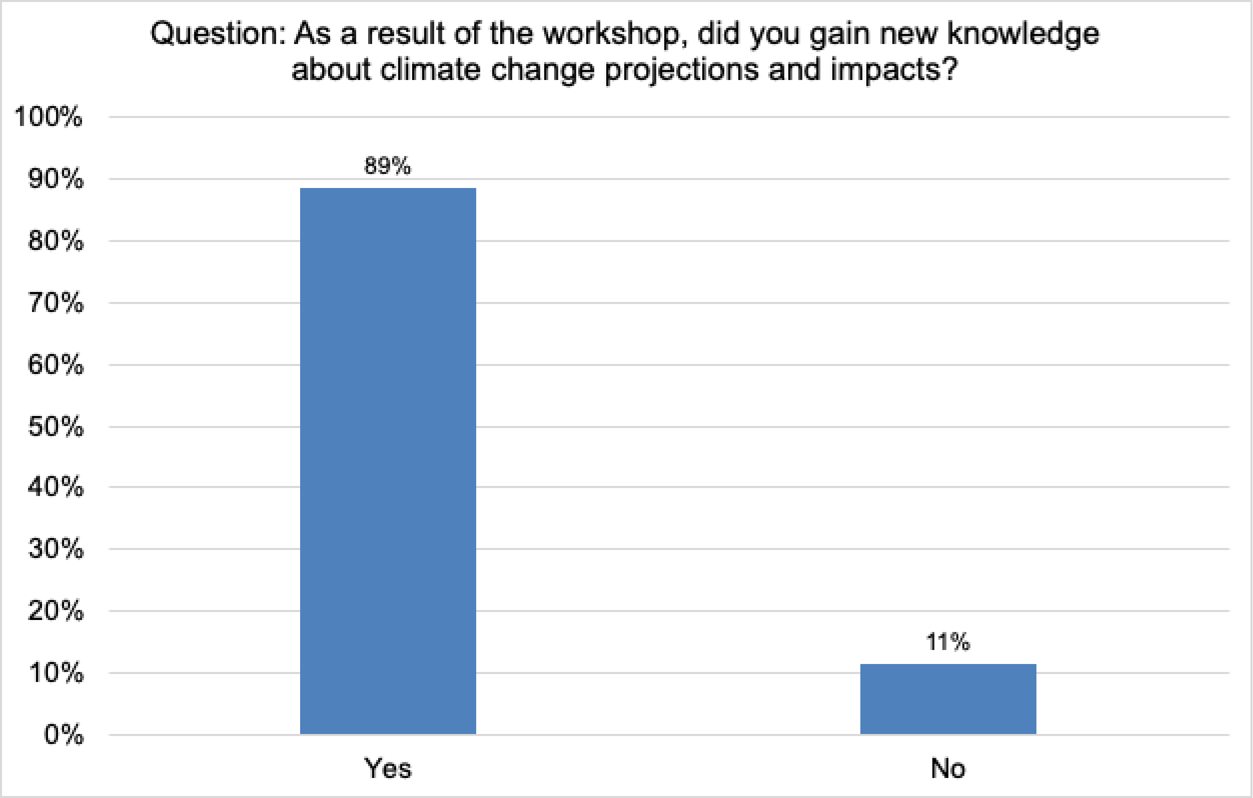
Question: As a result of the workshop, did you gain new knowledge about climate change projections and impacts?
100%
90%
80%
70%
60%
50%
40%
30%
20%
10%
0%
89%
11%
Yes
No

b)

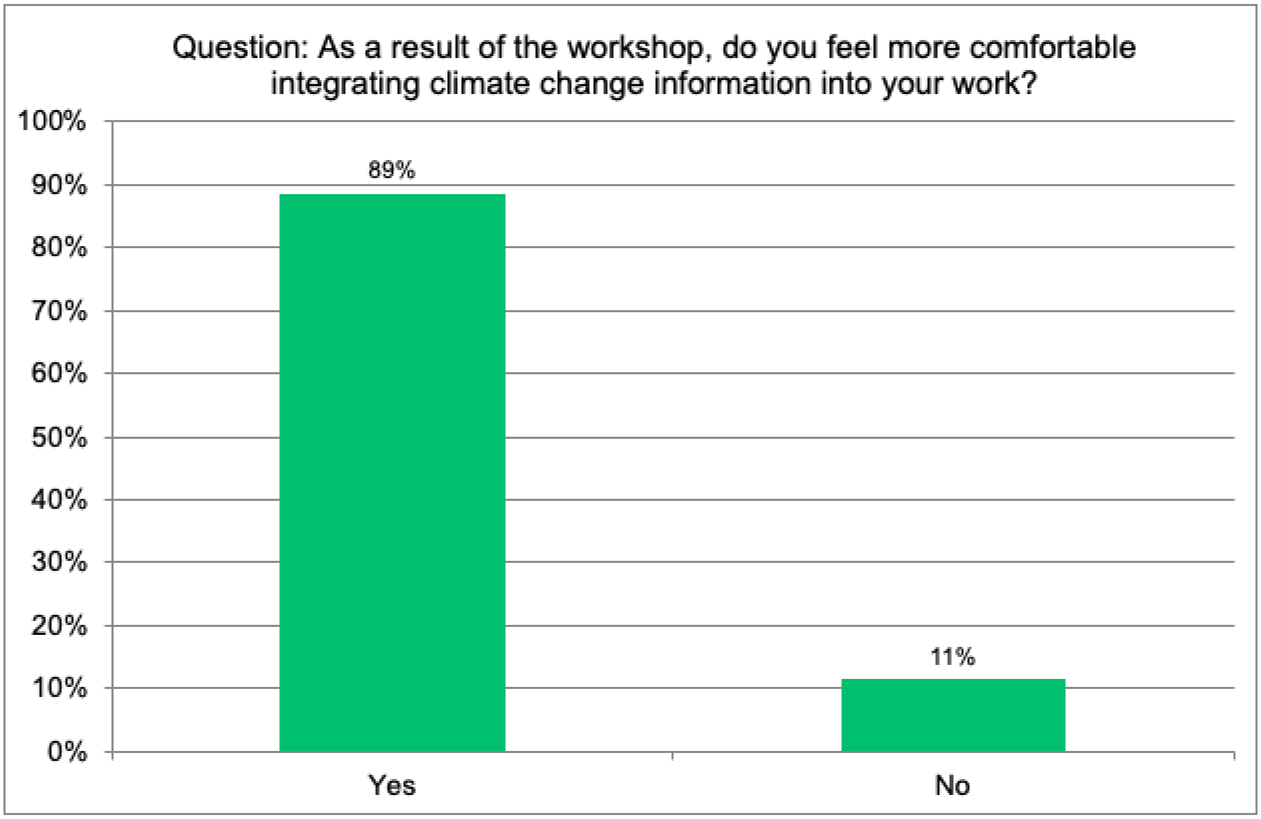
Question: As a result of the workshop, do you feel more comfortable integrating climate change information into your work?
100%
90%
80%
70%
60%
50%
40%
30%
20%
10%
0%
89%
11%
Yes
No

c)

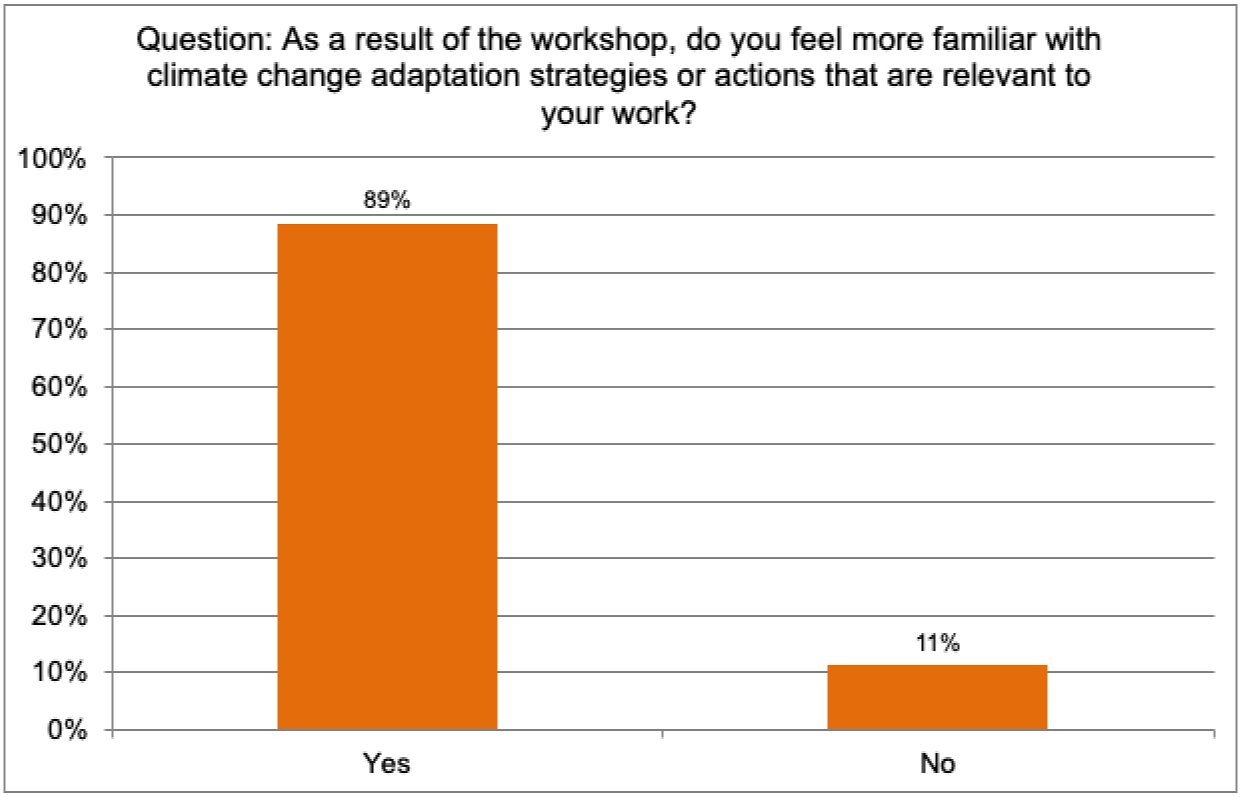

Fig S1 Results from a post-workshop survey that asked agency participants whether, as a result of the workshop, they: (a) gained new knowledge about climate change and its impacts, (b) felt more comfortable integrating climate change into their work (b), and (c) felt more familiar with climate change strategies and actions that are relevant to their work (n=35).

The inclusion of an optional unique identifier on both the pre-workshop survey (final question) and post-workshop survey (first question) allowed us to compare individuals' responses to the same questions immediately before and after the workshop. This analysis used data from three 11-point, likert-scale questions that asked agency participants: 1) how knowledgeable are you about climate change projections and impacts as they relate to your work?, 2) how comfortable are you with integrating climate change information into your work?, and 3) how familiar are you with climate change adaptation strategies or actions that are relevant to your work? Likert-scale responses ranged from 0-10, where 0 indicated not at all knowledgeable, comfortable, or familiar, and 10 indicated very knowledgeable, comfortable, or familiar. A total of 27 agency participants provided a unique identifier and answered the likert-scale questions both before and after the workshop. We used this data to examine whether a participant's level of knowledge, comfort, or familiarity with the topic going into the workshop affected whether and to what degree that participant indicated a change in those levels after the workshop by conducting a linear regression to compare an individual's response on the pre-workshop survey and the amount of change that same individual indicated on their post-workshop survey (i.e., amount of change = post-workshop value minus pre-workshop value). Finally, we conducted a Welch's two-sample t-test to examine whether the pre-workshop likert responses were significantly different from the post-workshop likert responses for each of the three questions, for those individuals who provided a unique identifier (n=27).

Regression analyses indicate that agency participants who came into the workshop with relatively low self-reported knowledge, comfort, or familiarity with the topic gained the most (Table S1, Figure S2). Those who came into the

workshop already knowing a fair amount, feeling fairly comfortable, and being relatively familiar with the topic reported less of a change after the workshop.

A few individuals reported a slight negative change in their likert responses after the workshop. This was most notable for the question "How comfortable are you with integrating climate change information into your work?", for which 8 individuals indicated a lower comfort level after the workshop than before (Figure S2b). This result is reflected in the t-test results, which showed that the mean post-workshop response to the likert question about comfort was not significantly different from the mean pre-workshop response (Table S1). On closer examination, survey data reveal that despite reporting a negative change in their likert responses, 6 of those 8 individuals answered "yes" to the question "As a result of the workshop, do you feel more comfortable integrating climate change into your work?". Negative change in likert scores for those individuals could have been be due to them evaluating their comfort level somewhat lower after the workshop either because they didn't remember what they previously marked on the pre-workshop survey or because, although they feel more comfortable in general, the complexities of integrating climate change may have become more evident throughout the workshop. Thus, they ranked their comfort lower. The largest negative change in response to the comfort question (-5) was from a respondent who did not attend any of the interactive workshop sessions, which is when participants spent the most time discussing the consequences of climate changes for their work. That same individual is responsible for one of the two negative change responses for the other likert-scale questions (related to knowledge of climate changes and impacts, and familiarity with adaptation strategies and actions) (Figures S2a and S2c). The second negative change responses for the knowledge and familiarity questions were from respondents who also indicated "yes" to the question about whether, as a result of the workshop, they increased their knowledge or familiarity.

Table S1. Linear regression and Welch's two sample t-test results for three likert-scale survey questions, using paired survey responses from immediately before and after the workshop (n=27). For the linear regression, X-values are the pre-workshop likert response for an individual (higher value = greater knowledge, comfort, or familiarity); Y-values are the change in likert responses for that same individual (change = post-workshop value minus pre-workshop value). For the Welch's two-sample t-test, we compared mean likert responses on the pre-workshop and post-workshop surveys.

	**Linear Regression**				**Welch's Two-Sample t-test**		
**Survey Question**	**Slope**	**Intercept**	**Adjusted R^2**	**p-value**	**Mean pre-workshop**	**Mean post-workshop**	**p-value**
How knowledgeable are you about climate change projections and impacts as they relate to your work?	-0.84	6.08	0.62	<0.0001	5.37	6.93	0.00024
How comfortable are you with integrating climate change information into your work?	-0.89	5.87	0.65	<0.0001	5.70	6.48	0.15
How familiar are you with climate change adaptation strategies or actions that are relevant to your work?	-0.90	6.18	0.69	<0.0001	4.07	6.59	<0.0001

a)

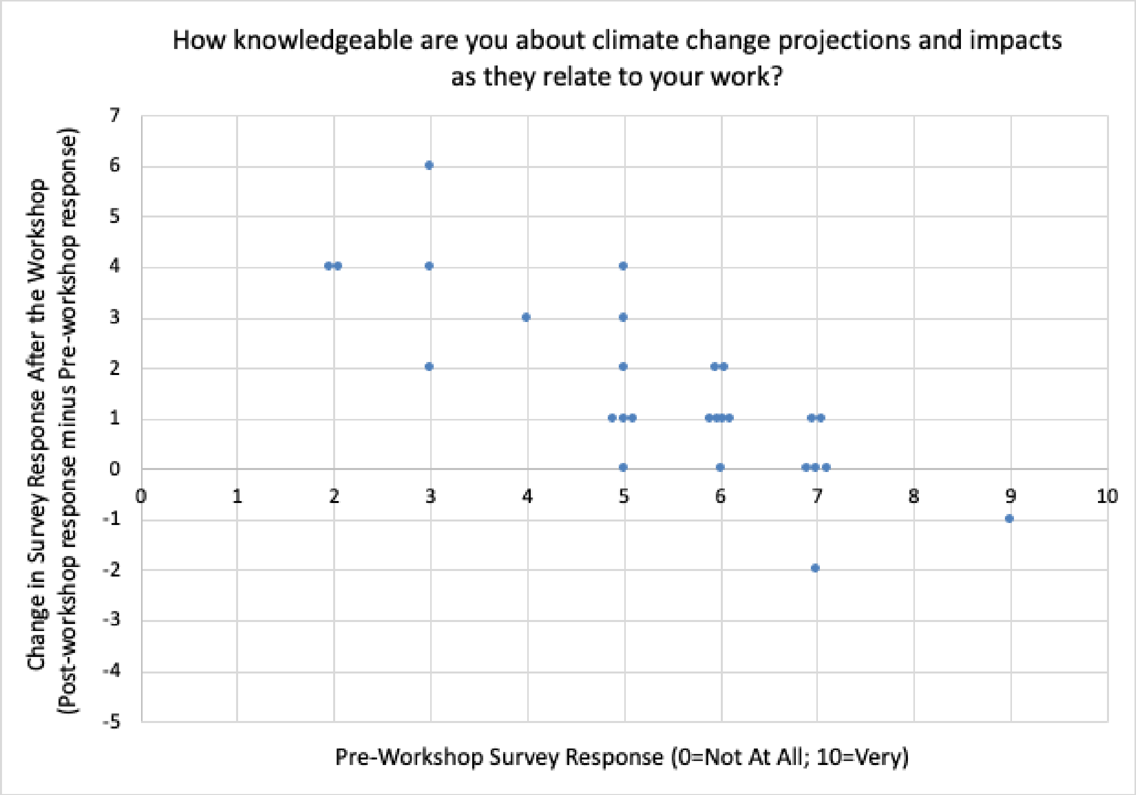
How knowledgeable are you about climate change projections and impacts as they relate to your work?
Change in Survey Response After the Workshop (Post-workshop response minus Pre-workshop response)
Pre-Workshop Survey Response (0=Not At All; 10=Very)

b)

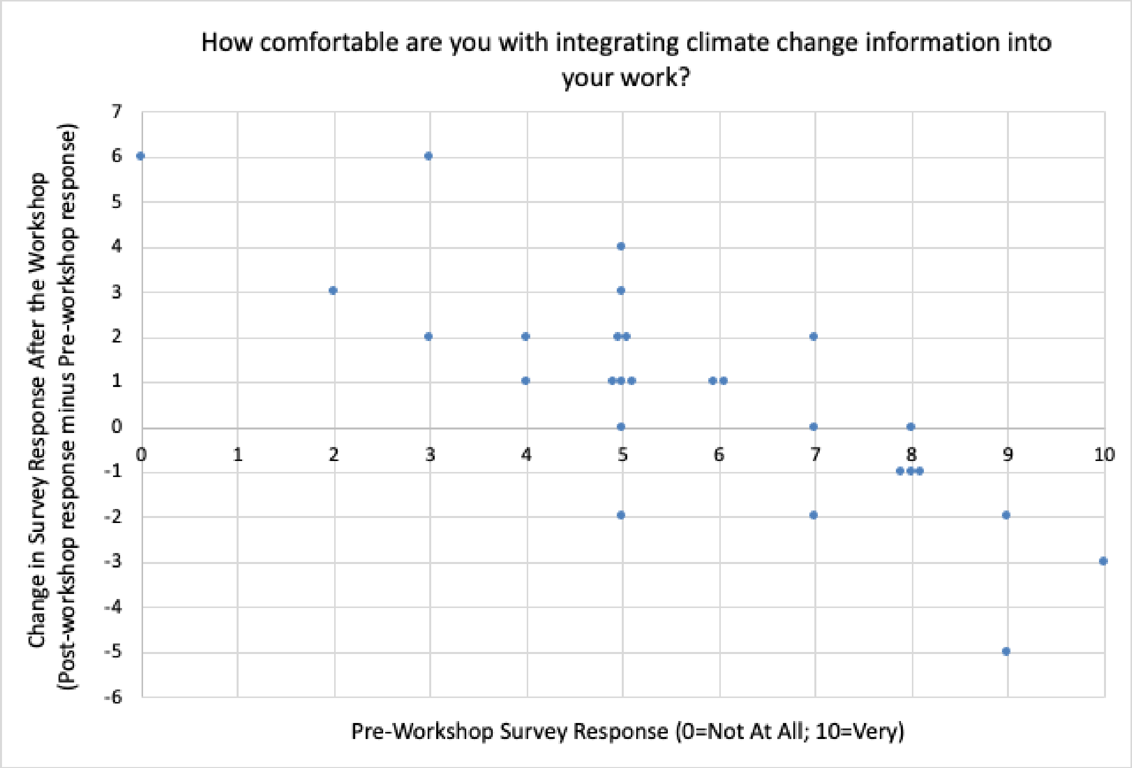
How comfortable are you with integrating climate change information into your work?
Change in Survey Response After the Workshop (Post-workshop response minus Pre-workshop response)
Pre-Workshop Survey Response (0=Not At All; 10=Very)

c)

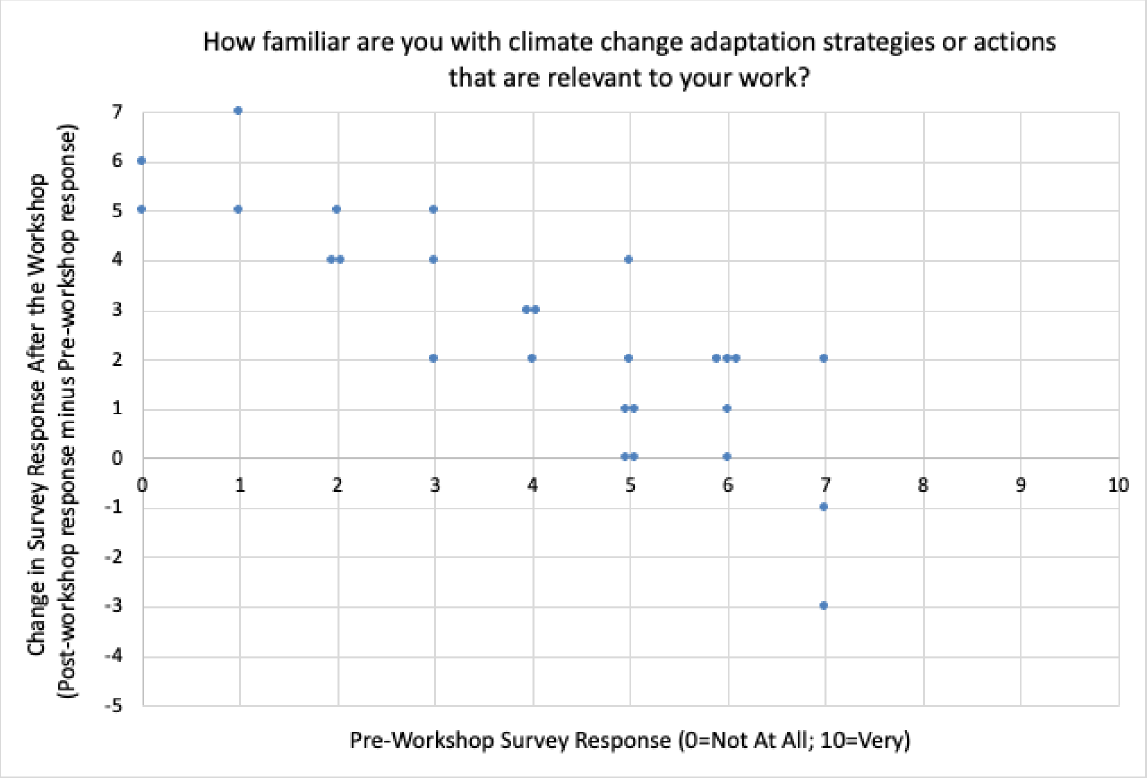

Fig S2 Relationship between an individual's response to three likert-scale questions immediately before and after a participatory workshop: (a) How knowledgeable are you about climate change projections and impacts as they relate to your work?, (b) How comfortable are you with integrating climate change information into your work?, and (c) How familiar are you with climate change adaptation strategies or actions that are relevant to your work? X-values are the pre-workshop likert response for an individual; Y-values are the change in likert responses (post-workshop response minus pre-workshop response) for that same individual. Overlapping points are shown with a small added displacement factor along the X-axis for display purposes only. Linear regression equations and significance values are shown in Table S1.

4.4 Post-Project Semi-Structured Interview Protocol

Recruitment email:

As part of evaluating outcomes from the collaborative project between Molly/WCS and the Wyoming Fish & Game Department to support the integration of climate change into the Statewide Habitat Plan (SHP), **we would like to schedule an hour-long interview with the core members of the Habitat and Technical Advisory Group that worked most closely on the project this past year.**

In the interview, we will ask you questions about the process for updating the SHP and how climate change was integrated into the plan. We will use the results of the interviews to inform our understanding of how management agencies integrate climate change considerations into management documents. In addition, we will explore what worked well about the project, and ways the process could have been improved. We will integrate these findings into a peer-reviewed journal article documenting the process. The interviews will be recorded. Your identity will be kept anonymous in any summaries of this work; however, given that the case study will focus on management planning by WGFD, it may be possible for readers to infer your identity based on your specific role within the agency.

Participation in the interview is completely voluntary. **If you are willing to participate, we would like to schedule the 1-hour interview between January 4-12. Please visit this link to write your name in your preferred slot on those days, at either 1:30 or 3:00 pm MT.**

Thank you so much for engaging in the process to work with Molly/WCS on integrating climate change into the SHP. Please let us know if you have any questions.

Interview script:
Thank you so much for agreeing to meet with us today. As we mentioned in the email the purpose of the interview is to evaluate the process used to integrate climate change into the 2020 Statewide Habitat Plan (SHP). We will start with background questions, then move to questions specific to the content of the 2020 SHP, and end with a few questions about the revision process.

As mentioned in the email, we are planning to record the interview for note-taking purposes - is it ok if we start that recording now?

[Block 1: Background and participation in Statewide Habitat Planning]

1. What is your title?

2. How long you have been working with the Department and a little bit about your job and responsibilities.

3. How did you first become involved in the drafting of the Statewide Habitat Plan for Wyoming?
    - Were you involved in the 2015 SHP? *(if yes be sure to ask probing questions below)*

4. What motivated you/the Agency to include climate change in the 2020 revision?
    - [IF YES above to 2015 involvement] What were the main factors that account for the difference in how climate change was included in 2020, relative to 2015?

Let's shift now to some questions about the 2020 SHP.

[Block 2: 2020 SHP revision]

5. In the 2020 SHP, there are 3 goals compared to the 5 goals in the 2015 SHP. Please tell us the main factors that motivated the team to update those goals.
    - Was climate change a factor in reshaping your goals? If yes, tell us more - what aspects of climate change were important to this process?

6. With regard to the Habitat Project Development and Funding Section (pg 14), how did the idea for incorporating climate change into the scoring criteria come about?
    - How will you evaluate whether or not to allocate the additional point for addressing climate change?
    - In what ways (if any) do you think this new scoring will change the kinds of projects implemented over time?

7. How did you decide which climate-related strategies and actions to include the plan?
    - What led to the flagging of climate-informed strategies and actions?

Ok let's now talk about the process that you, Molly and the rest of the core team at WGFD used for incorporating climate change into the SHP.

[Block 3: Evaluating project activities and boundary actor role]

8. What is your overall impression of how the process influenced the ways that climate change was integrated into the 2020 SHP (i.e., including the April workshop, the post-workshop meetings and discussions, and the Information Needs Survey)?

9. How did the switch to virtual meetings and workshops influence the process?
    - Did you have more/less/same participation?
    - Did you have more/less/same time for this project then you would have because of COVID?

10. For the purposes of this research, we would refer to Molly as a boundary actor or someone who works across science and practice. In what ways, if any, did involvement of Molly, the boundary actor, in the revision process influence the inclusion of climate change in the 2020 SHP, and the identification of management-relevant information needs?

    - Follow-up prompts - how did Molly's (or the boundary actor's) involvement in {see list below} shape those outcomes?
        - the setting of project goals,
        - planning the workshop,
        - facilitating the workshop,
        - summarizing workshop results,
        - conducting the information needs survey,
        - writing workshop report,
        - incorporating climate change into SHP

11. If you had a chance to give advice to another state agency as they **revise a habitat or wildlife management plan** to consider climate change, what would you say?
    - Would you recommend a similar approach or suggest modifications to the one we used?

12. What advice would you give about approaches to **identifying management-relevant climate change information needs**?
    - Would you recommend a similar approach or suggest modifications to the one we used?

13. [OPTIONAL, if time permits] What next steps will your agency take to implement climate-related strategies and actions or address research needs identified in the 2020 SHP?
    - How likely or unlikely are you to engage in follow-up collaborations (with WCS specifically, other organizations, or with other experts) to fill information needs?
    - What support would you/your Agency need to take those next steps?

To integrate throughout:

**** Do you think the role Molly played, or this process in general, is reproducible for other agencies?*

**** What was the general sentiment about the workshop going into it?*
